# Light-Mediated Antibacterial Activity of Composites of Polypyrrole and Green Zinc Oxide Nanoparticles Synthesized using *Sarcomphalus joazeiro* Extract

**DOI:** 10.1021/acsomega.5c03456

**Published:** 2025-09-20

**Authors:** Milena L. Guimarães, Ricardo F. Ferraz, Raquel A. P. Oliveira, José Galberto M. da Costa, Débora Odília D. Leite, Mateus M. da Costa, Helinando P. de Oliveira

**Affiliations:** 1 Institute of Materials Science, 74373 Universidade Federal do Vale do São Francisco, Avenida Antônio Carlos Magalhães, 510 - Santo Antônio CEP, 48902-300 Juazeiro, Bahia, Brazil; 2 RENORBIO - Northeast Biotechnology Network, Universidade Federal Rural de Pernambuco (UFRPE), Recife, Pernambuco 52171-900, Brazil; 3 Natural Products Research Laboratory, 226206 Universidade Regional do Cariri, Coronel Antônio Luíz Street, 1161−Pimenta, Crato 63105-000, Ceará, Brazil

## Abstract

The preparation of zinc oxide nanoparticles and zinc oxide-polypyrrole (ZnONPs@PPy) composites can be considered a critical step for enhancement in the antibacterial activity of the synthesized material, established by its electrostatic interactions with the bacterial cell wall. Herein, the reported novelty is based on the biosynthesis of ZnONPs by *Sarcomphalus joazeiro* and their subsequent incorporation into a composite with polypyrrole for impregnation into cotton fibers, enabling their use in wound dressing applications. The samples were characterized using HPLC, FTIR, SEM, TEM, XRD, and UV–visible spectroscopy, with the band gap determined by the Tauc method. HPLC analysis confirmed the presence of phenolic compounds in the extract applied to reduce ZnONPs. At the same time, FTIR indicated the presence of specific bonds of Zn–O in the synthesized ZnONPs. XRD assays indicated a hexagonal structure of ZnO, with crystallites of 46.69 nm, and TEM showed a distribution of particles with diameters ranging from 12 to 38 nm. By comparison, the UV–vis spectrum confirmed a plasmon band at 368 nm and a reduction in the band gap from 2.97 to 2.88 eV in the composite (ZnONPs@PPy). Antibacterial tests against *Escherichia coli* and *Staphylococcus aureus* demonstrated good efficacy of compounds under sunlight exposure, indicating that the smaller band gap width favored the interaction of ZnONPs and PPy with bacterial membranes, with the release of reactive oxygen species enhancing the antimicrobial action, resulting in significant bacterial cell damage and killing.

## Graphical abstract

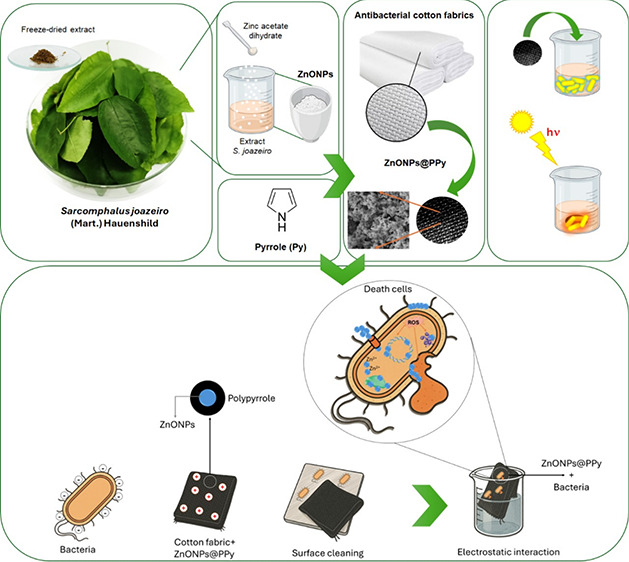

## Introduction

Advances in nanotechnology have led to innovations in several emerging fields, as evidenced by the diverse range of nanostructures produced, with different applications, from medicine to environmental remediation.^1,2^ Zinc oxide nanoparticles (ZnONPs) stand out due to their optical, electrical, and photocatalytic properties, as well as their high thermal stability and biocompatibility.^3,4^ Under UV light excitation, ZnONPs generate reactive oxygen species (ROS), which have been successfully used to degrade organic pollutants and inactivate microorganisms.^5^

These properties make ZnONPs promising prototypes for antibacterial applications. Although widely used in biomedicine, electronics, and environmental chemistry,^6–8^ ZnONPs are typically produced by physical or chemical methods that involve the use of toxic reagents, thereby raising concerns about environmental and health impacts.^9^ To overcome these limitations, the green synthesis of ZnONPs offers a sustainable alternative for large-scale compound production, utilizing ecologically benign materials such as plant extracts.^10,11^ In this process, the presence of biomolecules in the extracts reduces Zn^2+^ ions and stabilizes the nanoparticles, preventing agglomeration.^10,12^

The genus *Sarcomphalus* is comprised of species with recognized phytochemical potential, with *S. joazeiro* (Mart.) Hauenschild (previously classified as *Ziziphus joazeiro* Mart.^13^) is a promising species for green nanotechnology applications. Its leaves are rich in saponins and flavonoids, bioactive compounds that favor the synthesis of metallic nanoparticles through sustainable routes.^14,15^ *S. joazeiro* leaves and bark were previously characterized by UPLC-QDTF-MS/MS (Andrade et al.^16^), with detailed chemical profiling by Brito et al.^17^ and pharmacognostic characterization by Nascimento et al.^18^ Furthermore, other species of the genus have also been recognized as efficient stabilizing agents in the synthesis of ZnONPs.^19–21^

Moreover, conducting polymers, such as polypyrrole (PPy), are excellent supports for antibacterial applications and can be conveniently coated into nanoparticles.^22,23^ The resulting composite is favored by the intrinsic positive charge of polymeric chains of PPy that electrostatically interact with the negatively charged cell wall of bacteria. Bacterial cells’ attraction in the direction of the polymeric surface is a primary interaction mechanism, followed by ROS production. In terms of the biobased applications, Bengalli et al.^24^ reported that nanoparticles of PPy introduce negligible skin irritation, de Almeida et al.^25^ reported low cytotoxicity in PPy-based materials, and Ashour and Abd-Elhalim^26^ reported the safety of ZnO NPs obtained by green synthesis, Guo et al.^27^ highlighted that the combination of polypyrrole and zinc oxide (PPy/ZnO) is essential to improve the biocompatibility of magnesium alloys, favoring cell adhesion and proliferation, in addition to conferring high antibacterial activity (96.5 ± 2.6% against *E. coli*).

Herein, it is proposed (for the first time, to our knowledge) the green synthesis of ZnO by *S. joazeiro* followed by the production of composite with polypyrrole, exploring the direct polymerization of PPy on green ZnONPs and ZnONPs@PPy for deposition on cotton fibers, characterized as biocompatible and highly absorbent support that incorporates chemical properties of the composites, preserving the intrinsic properties of texiles for wearable applications.^28^

The modified cotton fibers and the composites were evaluated according to the resulting morphology and structure from SEM and TEM images, X-ray diffraction pattern, UV–visible absorption spectrum, and Fourier Transform Infrared Spectroscopy (FTIR), with the antibacterial evaluation from diffusion assays, kill time experiments, and antibiofilm activity against *S. aureus* and *E. coli*.

## Results and Discussion

### Phytochemical, Morphological, and Structural Analysis of *S. joazeiro* Extract and Reduced ZnONPs/ZnONPs@PPy Composites

The identification and quantification of secondary metabolites in *S. joazeiro* extract from HPLC were provided by comparison with reference standards of phenolic acids and flavonoids. Figure 1A presents the chromatographic profile of the analytical standards, indicating the retention times (*t*_R_) and the respective compounds.

**Figure 1:**
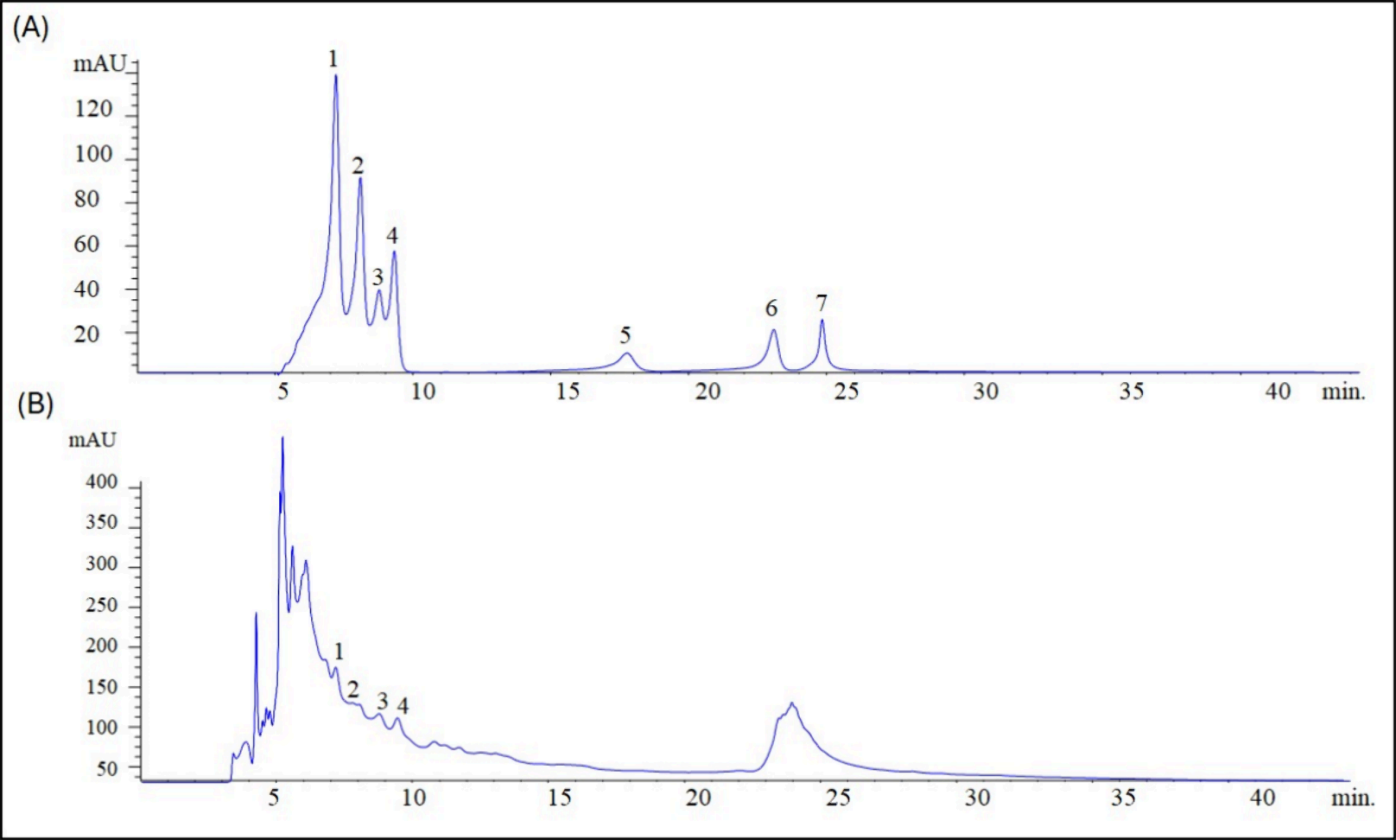
(A) HPLC profile of phenolic acids and flavonoid standards. Caffeic acid (*t*_R_ = 7.1 min, peak 1), p-coumaric acid (*t*_R_ = 8.0 min, peak 2), ferulic acid (*t*_R_ = 8.7 min, peak 3), cinnamic acid (*t*_R_ = 9.2 min, peak 4), naringenin (*t*_R_ = 17.7 min, peak 5), pinocembrin (*t*_R_ = 23.0 min, peak 6), and apigenin (*t*_R_ = 24.7 min, peak 7). Calibration curve for caffeic acid: *y* = 331.13*x* – 94.735 (*r* ^2^ = 0.9999); p-coumaric acid: *y* = 449.98*x* – 72.528 (r^2^ = 0.9971); ferulic acid: *y* = 185.3*x* −675.79 (*r* ^2^ = 0.9887); cinnamic acid: *y* = 236.74*x* – 188.67 (*r* ^2^= 0.9991); naringenin: *y* = 151.64*x* + 8.5796 (*r* ^2^ = 0.9994); pinocembrin: *y* = 177.66*x* – 390.72 (*r* ^2^ = 0.9955), and apigenin: *y* = 164.9*x* – 688.69 (*r* ^2^ = 0.9963). (B) HPLC profile of the aqueous extract of *S. joazeiro* leaves with the identification of caffeic acid (peak 1), p-coumaric acid (peak 2), ferulic acid (peak 3), and cinnamic acid (peak 4).

The results presented in Table S1 (data corresponding to Figure 1B) indicate that the prevailing phenolic compounds in the extract are caffeic acid (0.0233 ± 0.0029 mg/g), p-coumaric acid (0.0526 ± 0.0162 mg/g), ferulic acid (0.2410 ± 0.0156 mg/g), and cinnamic acid (0.0913 ± 0.0012 mg/g).

The identified phytochemicals play a key role in reducing and stabilizing ZnONPs. The green synthesis of these nanoparticles takes place through the reduction of Zn^2+^ ions present in the zinc acetate dihydrate solution, mediated by functional groups in the secondary metabolites of the extract, since flavonoids and phenolic acids act as electron donors, promoting the reduction of zinc ions to the zerovalent form (Zn^0^), followed by controlled oxidation to ZnO^29–32^ with the antioxidant compounds preventing uncontrolled oxidation, ensuring nanoparticle formation is homogeneous and stable.^33^ Figure S1 summarizes a scheme involving the interaction of ferulic acid and zinc acetate in the overall reduction process to ZnO nanoparticles. In this scheme, the thermal treatment introduces a critical step in forming the final product, which agrees with data reported in the literature.^34^

In general, the mechanism of ZnO nanoparticle synthesis mediated by plant extracts takes place in a three-step process: (i) activation, (ii) growth, and (iii) termination. In the activation phase, Zn^2+^ ions are reduced and nucleated by the reducing compounds present in the extract.^35^ In the growth phase, phytochemicals interact with the nanoparticles, preventing agglomeration and favoring a more homogeneous morphology.^29^ At the termination step, stabilizing agents (capping agents) determine the final shape of the nanoparticles, affecting their physicochemical properties and stability.^36^

Following the characterization of the produced nanoparticles, Figure 2A shows the experimental XRD pattern of the ZnONPs sample powder compared with the ZnO reference standard (ICSD 76641),^37^ suggesting that the phases are equivalent.

**Figure 2:**
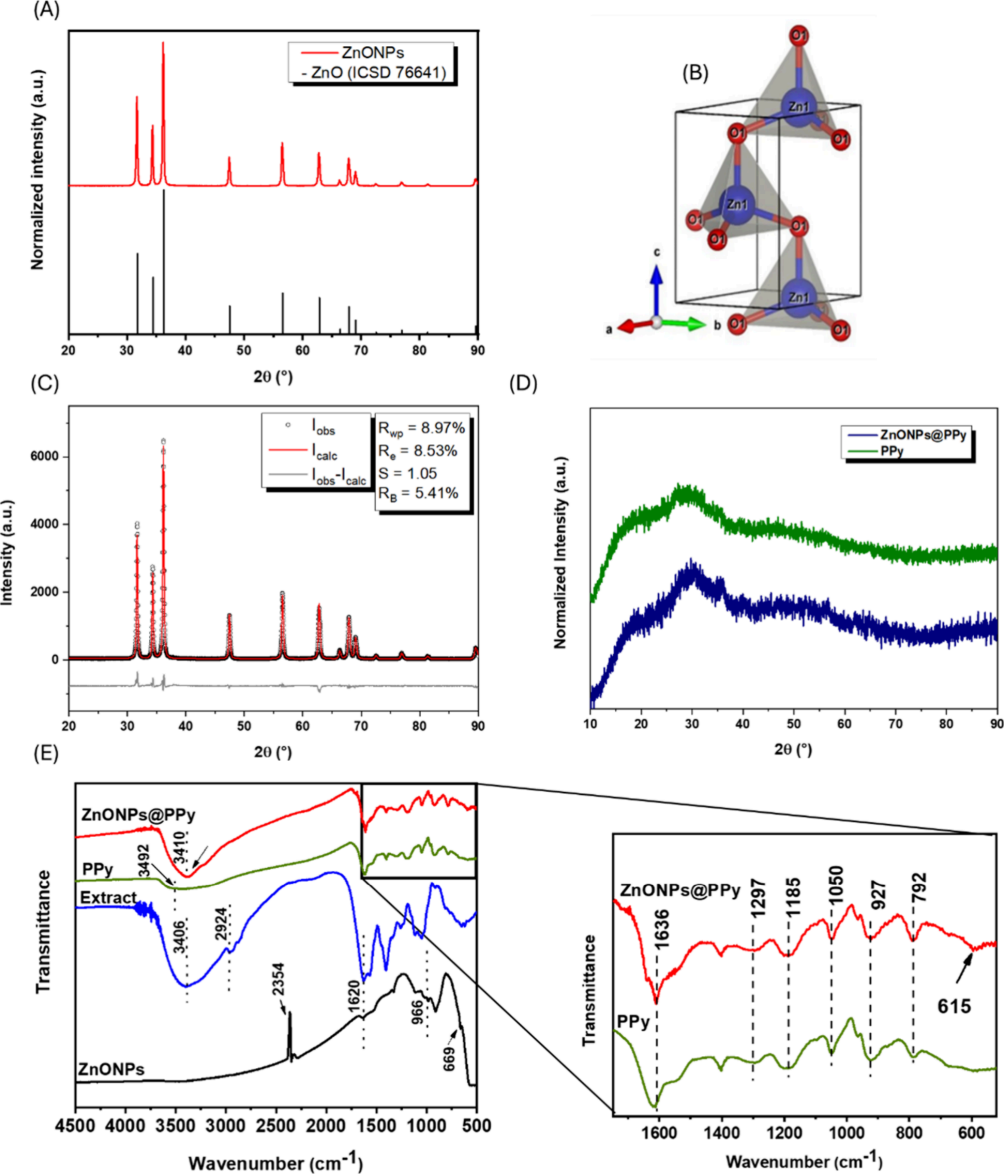
(A) Experimental XRD pattern of the ZnONP sample powder compared with the ZnO reference standard (ICSD 76641); (B) three-dimensional representation of the crystal structure of the ZnO reference (ICSD 76641); (C) observed intensities (*I*_obs_), intensities calculated by the Rietveld method (*I*_calc_), and the difference in intensities (*I*_obs_ – *I*_calc_) for the ZnONP sample and the refinement quality factors; (D) experimental XRD patterns of the PPy and ZnONPs@PPy sample powder; (E) FTIR spectrum of the analyzed samples: lyophilized extract of *S. joazeiro* leaves (curve in blue), zinc oxide nanoparticles in powder form (ZnONPs, curve in black), polypyrrole (PPy, curve in green), and ZnONPs composite with PPy (ZnONPs@PPy, curve in red).

The three-dimensional representation of the crystal structure of the ZnO reference (ICSD 76641) is shown in Figure 2B. The crystal structure of ZnO corresponds to a hexagonal system with space group P63mc (no. 186). The unit cell parameters are *a* = 3.250 Å, *b* = 3.250 Å, *c* = 5.207 Å, with angles α = 90°, β = 90°, and γ = 120°, resulting in a unit volume of 47.61 Å^3^ and a density of 5.68 g·cm^–3^. In the wurtzite structure of ZnO, each zinc ion (Zn^2+^) is tetrahedrally coordinated to four oxygen ions (O^2–^). Each Zn^2+^ ion forms partial covalent bonds with four O^2–^ anions, creating a stable three-dimensional lattice typical of wurtzite-type structures.

The experimental patterns were adjusted to the theoretical standard for ZnO (ICSD 76641) using Rietveld refinement for a quantitative evaluation. Figure 2C shows the experimental XRD pattern of the ZnONPs sample powder and the overlaid fitting by Rietveld refinement concerning the theoretical standard for ZnO (ICSD 76641). The results indicated a satisfactory adjustment between the observed and calculated intensities. The difference between the experimental values and those calculated by the model (*I*_obs_ – *I*_calc_) was minimal, suggesting accuracy between the experimental data and the reference structure of ZnO.

The refinement quality factors, which establish the residuals or deviations between the experimental and calculated values, are also presented in Figure 2C. The quality of the Rietveld refinement is quantified by the profile factor (*R*_p_), the weighted profile factor, or obtained error (*R*_wp_), and the minimum error, or expected error (R_e_). The ratio of *R*_wp_ to *R*_e_, represented by *S*, is a crucial parameter; a value of S close to 1 indicates a good fit, where *R*_wp_ is approximately equal to *R*_e_. Values of *S* ≥ 1 are considered plausible, with *S* < 1.5 generally regarded as satisfactory. The Bragg integrated intensity (*R*_B_) factors are also reported to assess the discrepancy between the observed diffraction peak intensities and those calculated by the model.^38^ The error values obtained (*R*_wp_ = 8.97%) and expected error (*R*_e_ = 8.53%) are acceptable for the method, resulting in a value close to unity of the refinement quality factor (*S* = 1.05). In addition, the Bragg integrated intensity index (*R*_B_ = 5.41%) was low, indicating the model’s adequacy.

The crystallite size is determined by the Scherrer formula (eq 1), where *D* is the crystallite size in nanometers, *K* is the Scherrer constant with a value of 0.9, λ is the wavelength of the X-ray source, equivalent to 0.15406 nm, β is the full width at half height (fwhm) in radians and θ are the peak positions in radians. The instrumental broadening correction of the peak width at half height (fwhm) values was previously applied for the calculations. As a reference, the parameters obtained by the Rietveld refinement were adjusted according to the equation of Caglioti et al.^39^ (eq 2). As a result, the crystallite size obtained for the ZnONPs sample was 46.69 nm.

D=(Kλ)/(βcosθ) 1

$$\beta^{2}=U\tan^{2}\;\theta +V\tan\theta +W$$ 2

For comparison with the response of the ZnONPs@PPy composite, a pure PPy sample was synthesized and evaluated in the DRX assays (see Figure 2D), indicating a typical amorphism, as expected. A broad peak was observed between 2θ = 15° and 35°, indicating the presence of disordered stacking of the polymer chains in the interplanar spacing. The high disorder inherent in the PPy matrix causes the XRD peaks to have a broad Gaussian shape.^40^ The pattern of the ZnONPs@PPy sample also showed predominant amorphism, with a broad peak observed between 2θ = 15° and 35°, characteristic of polypyrrole, as reported in the literature for corresponding systems.^41,42^

The FTIR spectrum of the leaf extract (Figure 2E, curve in blue) showed peaks at 3406, 2924, 1620, and 1404 cm^–1^. The peak at 3406 cm^–1^ corresponds to O–H stretching vibrations, indicating the presence of hydroxyl groups in plant extracts. The peak at 2924 cm^–1^ indicates C–H stretching vibrations of alkenes or methyl groups, while the peak at 1620 cm^–1^ is attributed to C=O stretching vibrations of carbonyl groups.^29^ The peak at 1404 cm^–1^ can be attributed to the symmetrical stretching of carboxyl groups present in amino acid residues of protein molecules.^30^

Zinc oxide nanoparticles (Figure 2E, curve in black) are characterized by a peak at 2354 cm^–1^, corresponding to the displacement of aliphatic stretching vibrations −CN that may be present in some alkaloid groups.^36,43^ The peak at 1620 cm^–1^ indicates the influence of proteins and enzymes involved in the stabilization process of the nanoparticles, which is attributed to the C=O stretching vibrations of the carbonyl groups.^29^ The peak at 966 cm^–1^ can be attributed to Zn–O stretching vibrations,^44^ while the peak at 669 cm^–1^ is assigned to the vibrations of ZnO produced by green synthesis.^43^

Regarding the interaction of ZnONPs with the polypyrrole (PPy) polymer, it was observed that the peak at 3410 cm^–1^ attributed to the N–H stretching in the pyrrole ring, which is shifted^45^ in the PPy sample (Figure 2E, curves in red and green). Characteristic peaks of polypyrrole were observed both in the pure PPy sample (Figure 2E, curve in green) and in the ZnONPs@PPy composite (1636, 1297, 1185, 1050, 927, and 792 cm^–1^). The peak at 1636 cm^–1^ corresponds to the C=C stretching vibration in the pyrrole rings, an unsaturated aromatic heterocyclic organic compound.^46^ The peak at 1297 cm^–1^ is associated with the vibration of the PPy rings.^47^ The peak at 1185 cm^–1^ can be attributed to the vibration of the pyrrole ring, while the peak at 1050 cm^–1^ is attributed to the C–H deformation. The peak at 792 cm^–1^ is associated with the =C–H vibration, while the peak at 927 cm^–1^ originates from the out-of-phase C–C.^46^ In addition, the appearance of the peak at 615 cm^–1^, characteristic of the Zn–O band,^48^ confirms the ZnO nanoparticles’ effective interaction with the polypyrrole structure.

The morphology of zinc oxide nanoparticles (ZnONPs), polypyrrole (PPy), and the composite (ZnONPs@PPy) was analyzed by scanning electron microscopy (SEM) and energy dispersive X-ray spectroscopy (EDS). Morphological analysis of zinc oxide nanoparticles (ZnONPs) indicated the high aggregation level induced by the sintering process with the formation of asymmetric clusters and irregular block shapes (Figure 3A), in agreement with the corresponding data reported in the literature.^49,50^ Figure 3B shows the overlaid EDS data on the SEM image, confirming the presence of the element zinc. The corresponding EDS map is shown in Figure S2.

**Figure 3:**
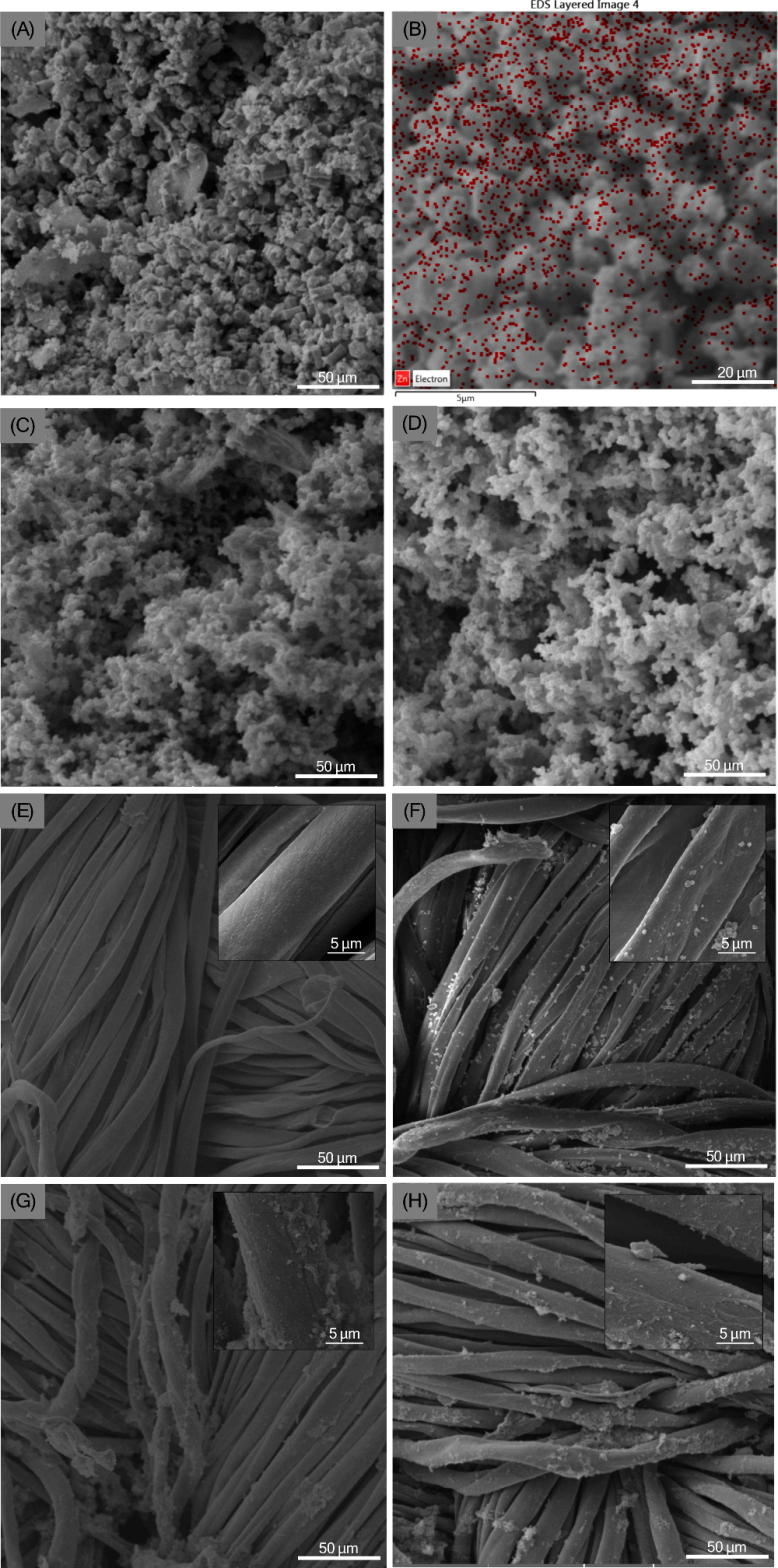
(A) Scanning electron microscopy (SEM) of zinc oxide nanoparticles (ZnONPs) powder; (B) energy dispersive spectroscopy (EDS) mapping of ZnONPs, with EDS image overlaid on the sample; (C) SEM image of polypyrrole (PPy) powder; (D) SEM image of ZnONPs@PPy powder; (E) SEM of the pure cotton fabric; (F) micrograph of the fabric coated with zinc oxide nanoparticles (ZnONPs); (G) micrograph of the fabric impregnated with polypyrrole (PPy); and (H) micrograph of the fabric impregnated with the ZnONPs@PPy composite.

The observed morphology of polypyrrole (PPy) as a grain-shaped structure (Figure 3C) is characteristic of chemical polymerization^51^ is preserved for the ZnONPs@PPy composite (Figure 3D), with the EDS analysis confirming the presence of Zn element in the structure. Figure 3E–H shows the SEM images for pure and impregnated cotton textiles with compounds.

As shown in Figure 3E, pure cotton fibers appear as microfibrils with a ribbon-like structure. Under the incorporation of ZnONPs by an ultrasonic bath-assisted immersion and drying method, it is possible to identify particle agglomerates on the surface of the cotton fibers (Figure 3F), in agreement with Nivedha et al.,^35^ and Subramanian et al.^52^ As reported by Lima et al., the fabrics coated with pyrrole (Figure 3G) showed a homogeneous coating deposition on the cotton fibers with some polypyrrole agglomerates.^28^ The fabrics modified with the ZnONPs@PPy composite (Figure 3H) showed a similar morphology to the previous case, as reported by Abdi et al.,^48^ for the incorporation of ZnO: Nd in the PPy nanostructures. The EDS analysis confirmed the presence of the Zn element in the fabric coated with ZnONPs.

Transmission electron microscopy (TEM) analysis was conducted to provide complementary information on the morphology, shape, and size of the ZnO nanoparticles. The results shown in Figure 4A confirmed the presence of agglomerates, resulting in particle overlap, with variations in the apparent thickness and contrast intensity in the micrograph, leading to areas of opacity (Figure 4B). In addition, it was identified that some particles exhibit irregular and varied shapes, in agreement with ref 53 Figure 4C shows the selected area electron diffraction (SAED) pattern used in the TEM of the ZnONPs. The size of the ZnONPs varied in diameter between 12 and 38 nm, with an average diameter of (24.47 ± 6.80) nm, as shown in the inset of Figure 4A, which is consistent with previous studies demonstrating the spherical shape formation of ZnONPs, as Mongy and Shalaby^54^ reported.

**Figure 4:**
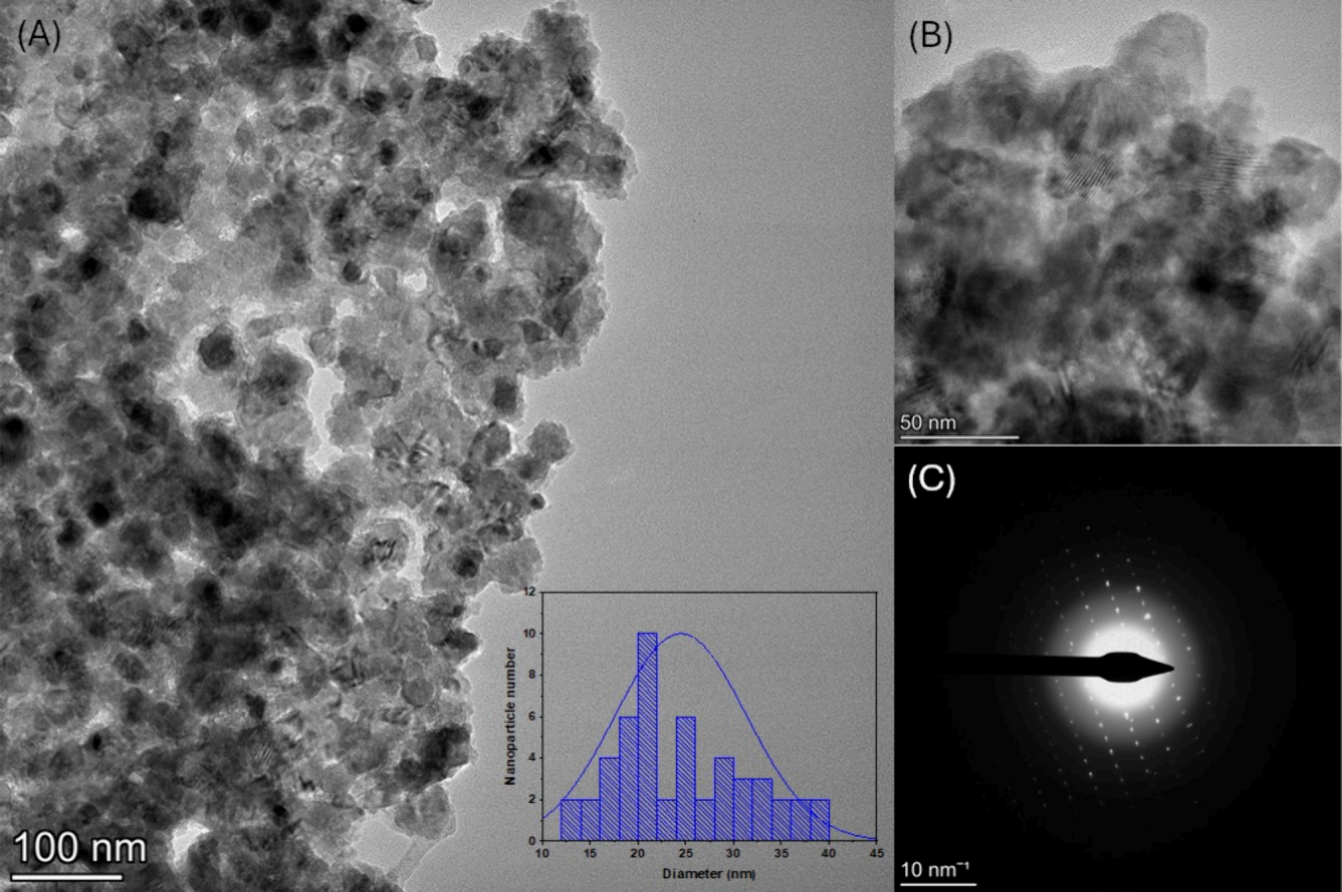
(A) TEM micrograph of zinc oxide nanoparticles (ZnONPs); (B) TEM micrograph of ZnONPs; and (C) selected area electron diffraction (SAED) pattern of ZnONPs.

DLS analysis returned a uniform size distribution and lower polydispersity (PDI = 0.266) for ZnONPs@PPy composite in comparison to ZnONPs (PDI = 0.451) and PPy (PDI = 0.471). The size distribution (shown in Figure S3) results in a distribution centered at 458.1 nm for ZnONPs, while the PPy sample shows a bimodal distribution, centered at 190.1 and 955.4 nm. The composite presented a distribution centered at 534.6 nm, confirming that a significant aggregation level is observed for the resulting ZnONPs-based nanostructures in water.

The UV–vis spectrum of the extract of *S. joazeiro* leaves is characterized by absorption bands at 245, 270, and 326 nm (Figure 5A), while the biosynthesized ZnONPs exhibited a single absorption band at 368 nm (Figure 5B). This result agrees with the values reported for ZnONPs produced from different plant extracts, ranging from 360 to 375 nm.^29,30,43^

**Figure 5:**
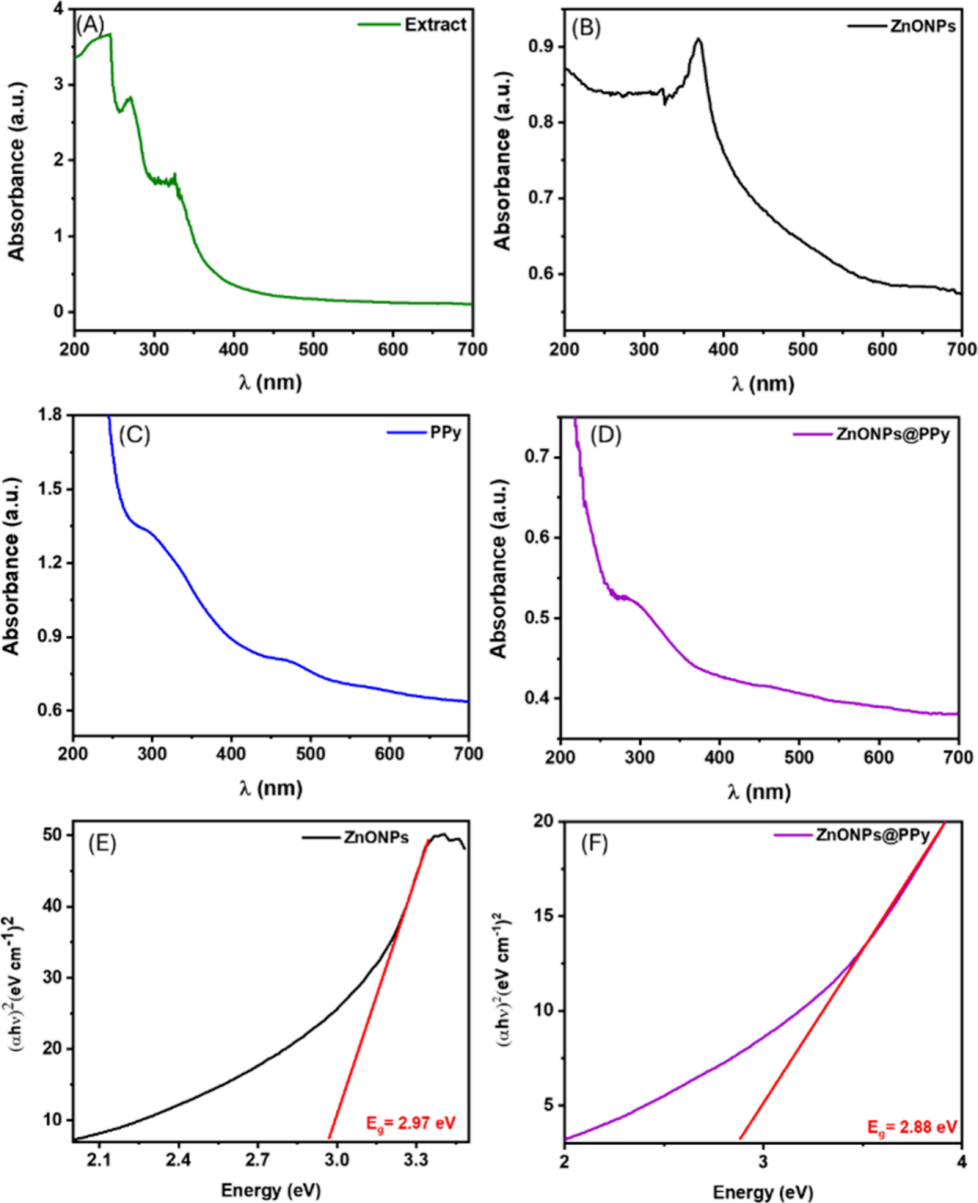
(A) UV–vis spectrum of *S. joazeiro* extract; (B) UV–vis spectrum of zinc oxide nanoparticles (ZnONPs); (C) UV–vis spectrum of polypyrrole (PPy); (D) UV–vis spectrum of ZnONPs@PPy composite; (E) band gap determination of ZnONPs; and (F) band gap determination of ZnONPs@PPy composite.

The samples PPy and ZnONPs@PPy were also characterized by UV–vis spectroscopy. Figure 5C shows the UV–vis spectrum of polypyrrole that exhibits an absorption band at 459 nm, assigned to the π–π* transition of polypyrrole,^55,56^ in correspondence with that observed in ZnONPs@PPy composite (Figure 5D).

The band gap energy of zinc oxide nanoparticles (ZnONPs) and the composite (ZnONPs@PPy) was determined using the Tauc method, which is detailed in the Supporting Information.^57^

The calculated band gap for ZnONPs was 2.97 eV (Figure 5E), corresponding to the value calculated for ZnONPs biosynthesized with *Calendula officinalis* leaf extract,^58^ which is lower than the conventional ZnO band gap, typically 3.3 eV.^59^ This behavior can be attributed to the interactions between the extract’s biomolecules and the NPs and possible structural defects in the crystal lattice.^44,60^

With the polymerization of PPy, a decrease in the composite’s band gap energy (ZnONPs@PPy) to 2.88 eV is observed (Figure 5F). This reduction may favor photocatalytic and antibacterial applications as it increases the reactivity of nanoparticles under UV–vis radiation, facilitating the generation of reactive oxygen species (ROS), which are harmful to bacterial cells.^58^ Figure S4 shows results for the corresponding photoluminescence of ZnO, confirming the characteristic peak from the exciting source. Both emissions (from ZnO synthesized by different methods (green and conventional ones)) are characterized by a sharp emission peak at 385 nm, which the literature attributes to the recombination of free excitons under collision events.^61,62^

### Antibacterial Activity of ZnO Nanoparticles, Polypyrrole, and ZnONPs@PPy Composite

The broth microdilution methodology was applied to determine the minimum inhibitory concentration (MIC) and minimum bactericidal concentration (MBC) of ZnONPs against *S. aureus* and *E. coli* bacteria. For *S. aureus*, the MIC and MBC values were 500 μg/mL and 1000 μg/mL, respectively. For *E. coli*, both the MIC and MBC were 2000 μg/mL, with results shown in Figure 6A,B.

**Figure 6:**
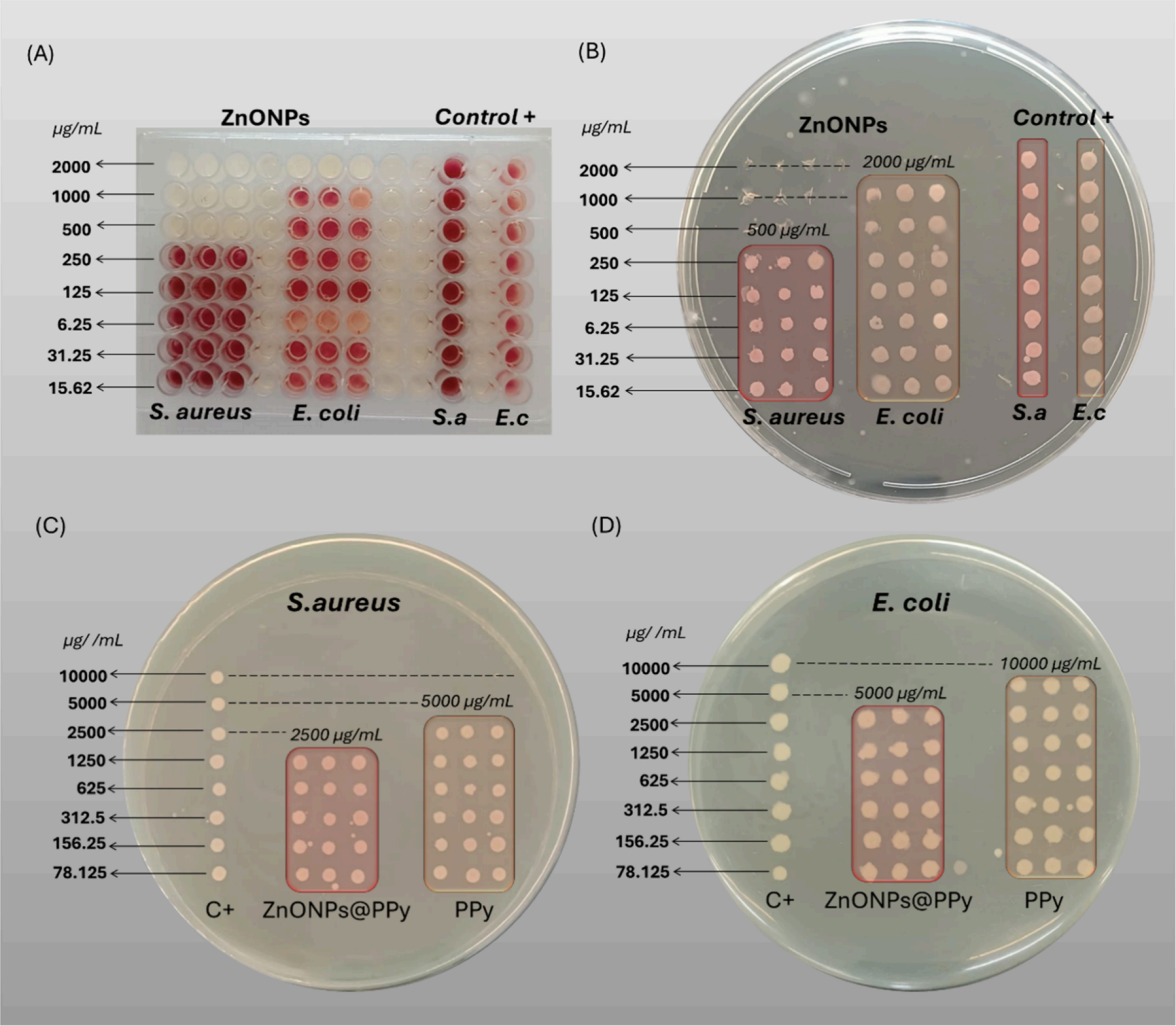
(A) Photo of 96-well microplate with the results of the minimum inhibitory concentration (MIC) assay of zinc oxide nanoparticles (ZnONPs) against *S. aureus* and *E. coli*; (B) petri dish showing the results of the minimum bactericidal concentration (MBC) assay of ZnONPs; (C) MBC result for polypyrrole (PPy) and ZnONPs@PPy composite samples against *S. aureus*; and (D) MBC result for PPy and ZnONPs@PPy samples against *E. coli*.

The superior activity of ZnONPs against Gram-positive bacteria compared to Gram-negative bacteria can be attributed to the structural differences in the cell walls and the interaction mechanisms with nanoparticles.^58^ Gram-negative bacteria have a thin peptidoglycan layer (2–3 nm) surrounded by an outer membrane composed of lipoproteins and lipopolysaccharides (LPS). This outer membrane creates a robust physical and chemical barrier, hindering the penetration of nanoparticles. In contrast, Gram-positive bacteria have a significantly thicker peptidoglycan layer (20–80 nm) but lack this additional lipid barrier, making them more susceptible to the action of ZnONPs.^29^

The association of ZnONPs and PPy is a strategic step to enhance the antibacterial properties of the nanoparticles. Broth microdilution assays revealed that the ZnONPs@PPy composite exhibited an MBC of 5000 μg/mL against *E. coli* and 2500 μg/mL against *S. aureus*, while pure PPy presented higher values (10000 μg/mL for *E. coli* and 5000 μg/mL for *S. aureus*), as shown in Figure 6C,D. These results suggest a synergistic interaction between ZnONPs and PPy, in which combining their physicochemical characteristics promotes an enhanced antimicrobial effect in the composite (the determination of MIC was not possible for pure PPy and the ZnONPs@PPy composite due to the dark coloration of these samples, which interfered with the detection of the color change promoted by triphenyl tetrazolium hydrochloride (CTT)).

To compare the antimicrobial profiles of green ZnONPs and conventional ZnONPs, a route with all analytical reagents was conducted (as described in the Supporting Information) with results summarized in Table S2. As can be seen, conventional ZnONPs (ZnONPs-C) are more effective against both strains tested. Against *S. aureus*, the lower MIC (250 μg/mL) indicates greater inhibitory capacity than green ZnONPs (MIC of 500 μg/mL), although both achieved the same MBC. Against *E. coli*, conventional ZnONPs were more effective, with lower MIC and MBC than the green synthesis. These data suggest that the conventional route, by generating particles with a higher purity degree and less interference from organic compounds, favors antimicrobial action, especially against Gram-negative bacteria. On the other hand, environmentally friendly processes present intrinsic properties of less aggressive components and the same order in the characteristic antibacterial concentrations.

Figure 7 presents the results of the time-kill kinetics tests of the three nanomaterial-based systems (ZnONPs, PPy, and ZnONPs@PPy) against *S. aureus* (Figure 7A,B) and *E. coli* bacterial cells (Figure 7C,D) conducted under sunlight exposure and dark conditions. The analysis was performed at fixed intervals of 0, 10, 20, 30, 60, 120, and 180 min, after which the number of viable cells in contact with the antibacterial samples was recorded. The tests were conducted using 2 × MBC against the respective bacteria. As can be seen, pure ZnONPs did not show a significant bactericidal effect, exhibiting a similar response to the negative control.

**Figure 7:**
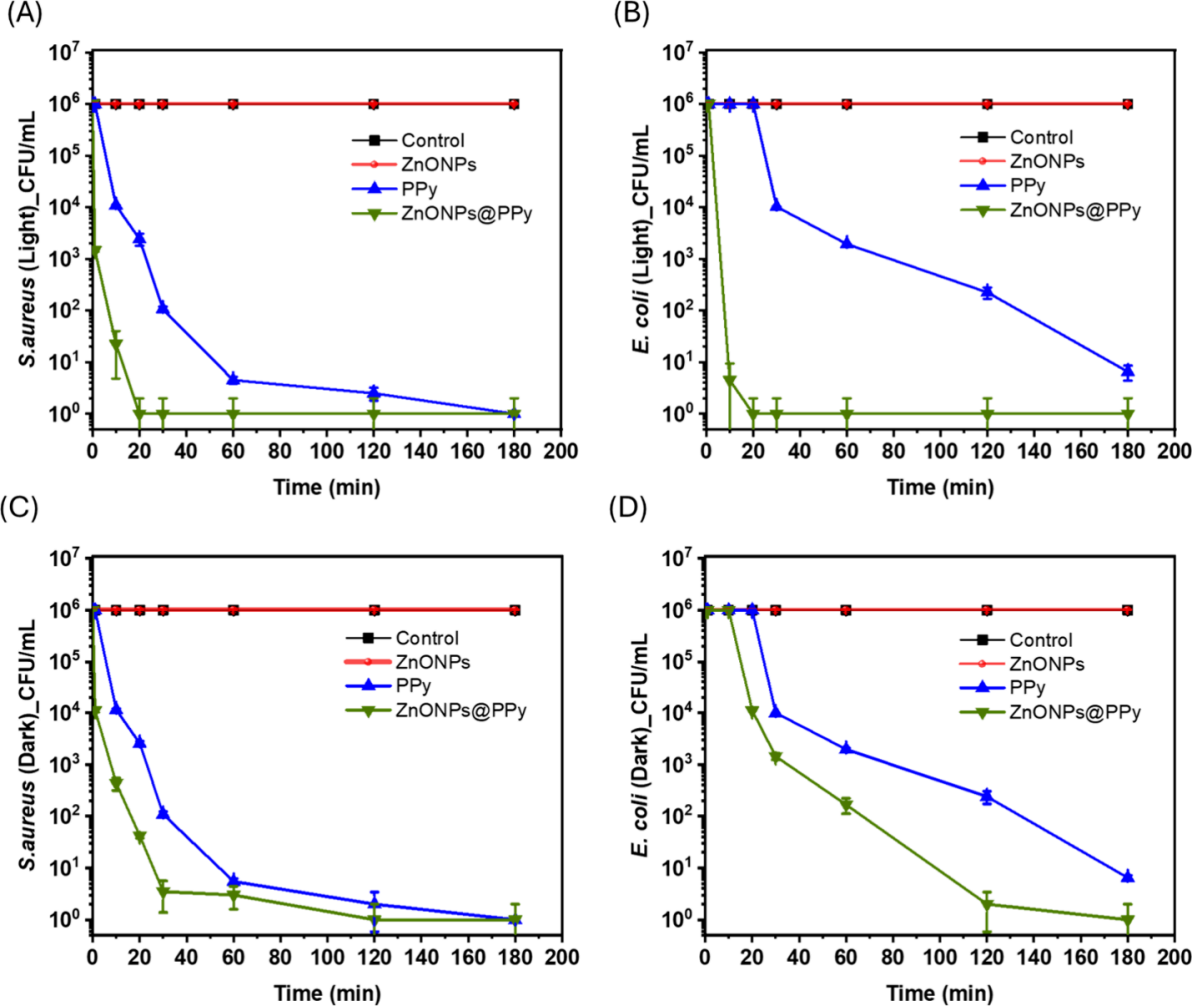
Bacterial killing kinetics over time, assessed by viable cell counting for the ZnONPs, PPy, and ZnONPs@PPy samples. The assay was conducted under sunlight (A, B) and dark (C, D) exposure conditions, allowing comparison of the effects of different experimental conditions on antimicrobial activity against *S. aureus* (A, C) and *E. coli* (B, D).

The pure polypyrrole samples showed moderate antibacterial activity, with variable efficacy for *E. coli* and *S. aureus*. For *E. coli*, the number of viable cells decreased progressively from 10^6^ CFU/mL to 10^1^ CFU/mL after 180 min. In contrast, *S. aureus* demonstrated a high degree of susceptibility to polypyrrole, significantly reducing colonies after 10 min of contact and eliminating viable colonies in 120 min. This difference between the species is in line with broth microdilution tests and previous studies^51^ which indicates that the structure of the cell wall of Gram-positive bacteria favors a more intense interaction with PPy, amplifying its antibacterial effect.

As expected, the antibacterial action of pure polypyrrole was not influenced by light since there was no significant variation in the number of viable colonies by comparison of systems under exposure to solar radiation and the dark environment. On the other hand, the ZnONPs@PPy composite demonstrated superior antibacterial performance compared to pure ZnONPs and pure polypyrrole. For *E. coli*, in a dark environment, the number of viable cells decreased after 20 min, with total eradication of colonies occurring within 120 min. Exposure to solar radiation intensified the antimicrobial action, resulting in a significant reduction in cell count after 10 min and complete elimination after 20 min. For *S. aureus*, the effect of the composite was even more pronounced. In the dark environment, bacterial viability was reduced within the first minute of contact, resulting in the total eradication of colonies within 120 min. Under exposure to light, the antimicrobial response was accelerated, eliminating viable cells after 20 min.

The kinetic tests reinforce that exposure to light enhances the antibacterial activity of ZnONPs@PPy against *S. aureus* and *E. coli*, evidencing the role of the photoinduced effect in the material’s performance. Under sunlight radiation, ZnONPs@PPy derivatives enhance the production of reactive oxygen species (ROS) in response to severe oxidative stress.^32,63,64^ This process triggers structural damage to essential biomolecules, including lipids, proteins, and DNA, thereby inhibiting bacterial cell replication and division.^30^

Combining the antimicrobial mechanisms of polypyrrole and ZnONPs explains the superior efficacy of the ZnONPs@PPy composite. While PPy destabilizes the cell membrane through electrostatic interactions, Zn^2+^ ions intensify oxidative damage, resulting in a highly efficient bactericidal effect. This combined approach highlights the composite’s potential for biomedical applications and the development of new advanced antimicrobial materials.^22,65^

The superior performance observed for samples excited with the sunlight can be explained as follows: with the reduction in the bandgap of the composite, the photoinduced process is favored, with ZnO absorbing UV light and generating electron–hole pairs (e^–^–h^+^). The holes are transferred to PPy, which interacts with adsorbed H_2_O molecules, forming oxidizing radicals (•OH). The PPy coating facilitates the migration of holes to the particle surface, reducing the recombination of e^–^–h^+^ pairs and improving the photocatalytic efficiency of the material, making it promising for environmental and antimicrobial applications.^66^ The combination of polypyrrole’s antimicrobial mechanisms explains the superior efficacy of the ZnONPs@PPy composite. The prevailing mechanisms of antibacterial activity in composites were evaluated according to the NBT method, with results shown in Figure S5. As can be seen, the negative control (a) and pure polypyrrole without bacteria (b) are compared with the positive control (H_2_O_2_) (c) and samples (ZnO (d), PPy (e), and ZnONPs@PPy (f)) immersed in culture media with *E. coli* and in the NBT dispersion. A negligible change in color in the halo is observed in the negative control. In contrast, diffusive ROS species are abundant in the positive control (changing the color of the agar in the well). While a negligible formation of ROS diffusive species halo is observed for ZnONPs, an increasing diameter for the composite (ZnONPs@PPy) compared with pure PPy, characterizes the ROS formation as a prevailing mechanism for PPy-based samples.

### Antibacterial Assays with Modified Textiles

To evaluate the efficiency of impregnated textiles with composites against bacteria, agar diffusion tests were performed using cotton fabric discs impregnated with ZnONPs, PPy, ZnONPs@PPy, and control discs (fabrics without adding antimicrobial agents). Each test was conducted in triplicate against the pathogenic bacteria *S. aureus* and *E. coli*.

For tests with impregnated fabrics, the concentration of ZnONPs had to be increased 10-fold due to absorption by the fibers. Thus, a 20 mg/mL concentration was established for the experiments involving textiles impregnated with ZnONPs.

The inhibition zone diameters showed higher efficacy for PPy-based and ZnONPs@PPy-modified fabrics than ZnONPs-impregnated fabrics, as shown in Figure 8. Against *E. coli*, the inhibition zone diameters were (5.5 ± 0.5) mm, (13.0 ± 0.8) mm, and (16.0 ± 0.8) mm for ZnONPs, PPy, and ZnONPs@PPy samples, respectively (as shown in Figure 8A,B). For *S. aureus*, the inhibition zone diameter values were (9.2 ± 0.2) mm, (15.2 ± 0.6) mm, and (18.0 ± 0.8) mm for the same sample sequence (Figure 8C,D). A positive control was performed with ciprofloxacin (0.1 mg/mL), and results for the corresponding zone of inhibition experiments are provided in Figure S6 (S6a against *S. aureus* and S6b against *E. coli*).

**Figure 8:**
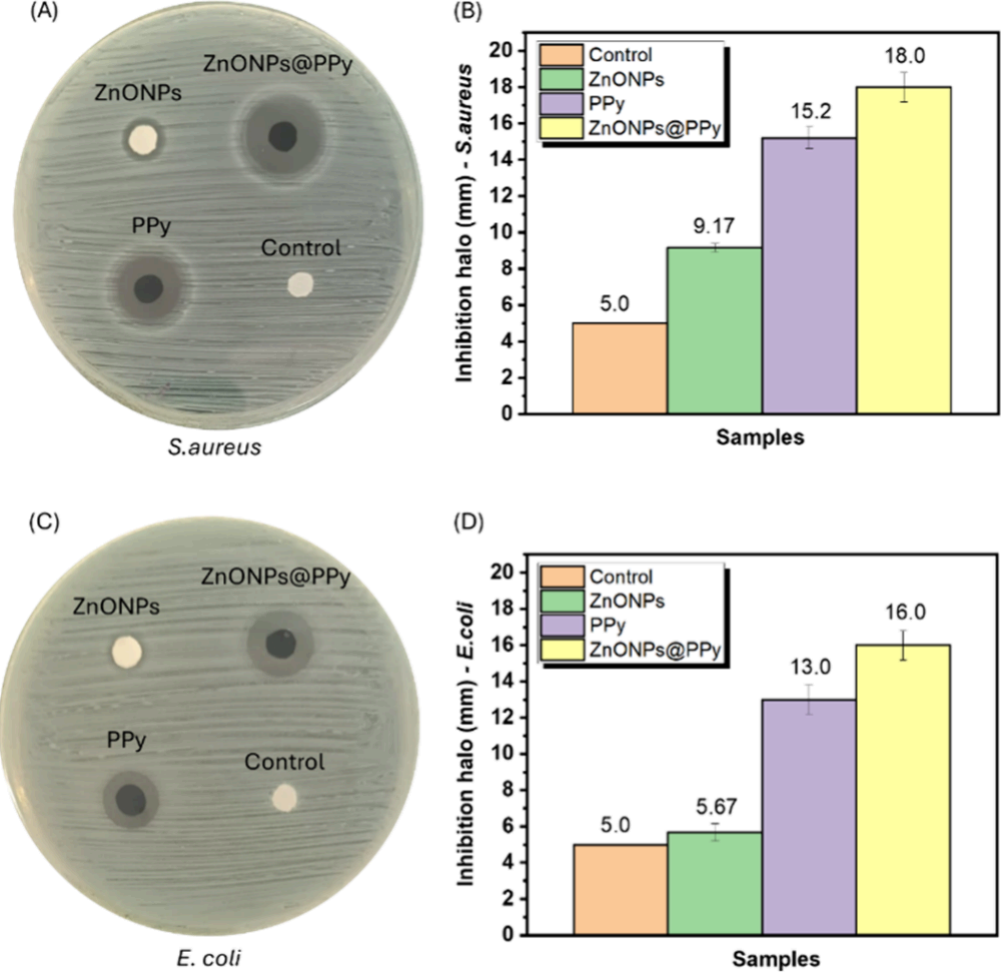
(A) Photo of the inhibition zones obtained for cotton fabric samples impregnated with ZnONPs, PPy, and ZnONPs@PPy, compared to the negative control against *S. aureus*. (B) Mean values of the diameters of the inhibition zones for *S. aureus* and the standard deviation. (C) Image of the inhibition zones obtained for cotton fabric samples impregnated with ZnONPs, PPy, and ZnONPs@PPy, compared to the negative control against *E. coli*. (D) Mean values of the diameters of the inhibition zones for *E. coli* and the standard deviation.

Previous work by the authors’ team^28^ confirmed the formation of inhibition zones for CNT-PPy samples. The authors attributed this effect to combining positively charged species in the polymer chains with chloride ion (Cl^–^) diffusion, which enhanced the bactericidal action. These findings are comparable to those of the present study, in which fabrics modified with ZnONPs@PPy demonstrated superior antimicrobial activity than those containing pure ZnONPs. Bizuayehu et al. reported the green synthesis of ZnONPs using *Rumex nervosus* Vahl leaf extract.^36^ with inhibition zones of 18.3 mm for *S. aureus* and 16.6 mm for *E. coli* at a concentration of 60 mg/mL of ZnONPs. The present study tested ZnONPs at a significantly lower concentration (20 mg/mL), yielding promising results in conjunction with PPy.

The qualitative evaluation of the antibacterial activity of the nanomaterial-impregnated fabrics (cleaning prototypes) was performed using a surface disinfection test on sterilized stainless-steel plates (5 × 5 cm). The results in Figure S7 demonstrate significant differences among the tested materials, indicating the following order of antibacterial efficacy: ZnONPs < PPy < ZnONPs@PPy. In the control group, the cleaning procedure was performed using pure cotton fabrics, with the surface remaining highly colonized and exhibiting numerous bacterial colonies. Impregnation with ZnONPs resulted in a moderate reduction in CFU of *S. aureus* and *E. coli*, while fabrics impregnated with PPy showed a more pronounced decrease in bacterial growth. Notably, the wipes modified with the ZnONPs@PPy composite were the most efficient, eliminating viable bacterial cells.

These findings indicate that ZnONPs@PPy composite enhances antimicrobial activity, resulting in a highly effective material for surface decontamination. The superior performance of ZnONPs@PPy suggests its applicability in self-decontaminating fabrics and other antimicrobial surfaces, making it a promising alternative for preventing the spread of pathogens.^67^

The antibiofilm activity assays were also evaluated for modified textiles. The results demonstrated high antibiofilm activity for both the fabric with PPy and the fabric with the ZnONPs@PPy composite, with both showing 99.99% efficacy in inhibiting *S. aureus* and *E. coli* biofilms, regardless of the experimental condition of light or dark (Figure 9A,B)—the spectrum of the sunlight radiation acquired along with the experiment is shown in Figure S8. This corresponding performance highlights the intrinsic efficacy of PPy-based materials in antibiofilm-based systems. Guo et al.^65^ developed a multifunctional polypyrrole/zinc oxide coating applied to biodegradable magnesium alloys. The *in vitro* investigation demonstrated that this coating significantly favors cell adhesion and proliferation and presents high antibacterial efficacy, reaching (96.5 ± 2.6)% inhibition against *E. coli*.

**Figure 9:**
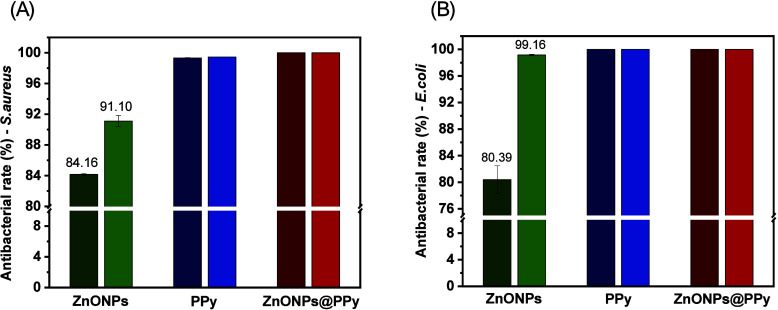
(A) Results for inhibition of biofilm formation of *S. aureus* (A) and *E. coli* (B) on cotton fabrics impregnated with ZnONPs, PPy, and ZnONPs@PPy. Quantification was performed by counting viable cells extracted by an ultrasonic bath, comparing conditions in the dark (dark bars) and under illumination (light bars).

On the other hand, the samples containing ZnONPs showed variations in the levels of inhibition of biofilm formation. For *S. aureus*, the inhibition rate was 84.16% in the dark, increasing to 91.10% under light exposure (Figure 9A). For *E. coli*, the increase was more significant, ranging from 80.39% in the dark to 99.16% under light (Figure 9B). These results reinforce that sunlight exposure affects the effectiveness of ZnO nanoparticles (ZnONPs). The outstanding antibiofilm activity of PPy makes the observed influence of the sunlight negligible on the overall response. Additional SEM images were performed to evaluate the cell adhesion on textiles. As shown in Figure S9, *S. aureus* cells were observed to be distributed along the surface of the pure cotton fabric. Similarly, in Figure S10A, *E. coli* cells adhered to the microfibrils of the pure fabric, demonstrating a scenario favorable to the formation of bacterial biofilms.

Figures S9B–D and S10B–D correspond to the fabrics impregnated with ZnONPs, PPy, and ZnONPs@PPy against *S. aureus* and *E. coli*, respectively, and show scarce bacterial cells on the modified surfaces.

The zeta potential values obtained for the polypyrrole (PPy), ZnONPs@PPy composite, and pure ZnONPs samples provide crucial information about the materials’ surface interactions with bacterial cells and insights into mechanisms for antibacterial activity. Polypyrrole presented a zeta potential of (+35.2 ± 1.0) mV, while the ZnONPs@PPy composite presented a value of (+30.8 ± 1.4) mV, and pure ZnONPs, in turn, had a negative value of (−11.6 ± 1.9) mV.

Although the presence of ZnONPs slightly reduced the positive charge of the ZnONPs@PPy composite (+30.8 ± 1.4 mV), the antimicrobial activity was enhanced since it remained positive (ζ > 30 mV). The antibacterial mechanisms of ZnONPs are incorporated to those of polypyrrole, providing a synergistic effect. The composite’s high electrostatic force facilitates the rupture of the bacterial cell membrane, promoting the destruction of anionic lipids.^46^ For comparison, Table S3 summarizes the properties and applications of ZnO nanoparticles reported in the literature and based on different synthesis routes.

Furthermore, the results obtained by UV–vis absorbance spectrum revealed the reduction of the band gap of ZnONPs from 2.97 to 2.88 eV in the ZnONPs@PPy composite, indicating a clear improvement in the photocatalytic potential compared to pure ZnONPs. As reported in the literature, ZnO has a high UV light absorption capacity and exhibits strong photoconductivity due to the generation of highly reactive oxygen species (O_2_^–^, O_2_^2–^) adsorbed on its surface. This process occurs in the surface electron depletion region, which is reduced or buffered after weakly bound oxygen species desorption under UV illumination.^59^ This change in the band gap suggests that the composite is more efficient in absorbing light and generating reactive species through the action of ZnONPs@PPy composite. Therefore, the results show that although pure polypyrrole demonstrates significant antibacterial activity, adding ZnONPs produced by green routes that optimize this action results in a composite with enhanced antimicrobial properties. The synergistic interaction of ZnONPs@PPy, as a promising material for antimicrobial applications, combines complementary mechanisms of action to improve its efficiency against pathogenic bacteria.

## Conclusions

This study evaluated the efficiency of *S. joazeiro* leaf extract in the green synthesis of ZnONPs and its association with polypyrrole in developing a new composite with relevant antimicrobial mechanisms. The material showed a high capacity to inhibit biofilm formation and eliminate planktonic bacteria, especially under exposure to sunlight. In addition to conferring stability to ZnONPs, the production of ZnONPs@PPy composite enhanced their antimicrobial action through photocatalytic and electrochemical mechanisms. The electrostatic interaction with bacterial membranes and the production of reactive oxygen species resulted in a material with superior activity than the isolated compounds. These findings highlight ZnONPs@PPy as a promising alternative for advanced antimicrobial applications, potentially applied in surface cleaning and bacterial removal.

## Materials and Methods

The experiments were carried out using zinc acetate dihydrate (Zn­(CH_3_CO_2_)_2_·2H_2_O), ferric chloride, and pyrrole (previously distilled and stored under refrigeration to prevent photodegradation and thermal oxidation), with all analytical-grade (P.A.) reagents acquired from Sigma-Aldrich. Fresh leaves of *S. joazeiro* were collected at the State University of Bahia, Juazeiro–BA, Brazil. Anhydrous ethyl alcohol (Prolink, Brazil) and ultrapure water with a resistivity of 18.2 MΩ.cm were used to prepare the solutions.

### Preparation of Aqueous Extract of *S. joazeiro* Leaves

Plant leaves were collected in Campus III of the Bahia State University (UNEB), Juazeiro, Bahia, Brazil. To ensure botanical authenticity, a voucher specimen was collected, herborized, and deposited in the Northeastern Sertão Reference Herbarium (HRSN), from the São Francisco Valley Biodiversity Center at Federal University of São Francisco Valley (CEBIVE/UNIVASF), under registry number HRSN 25443. The species was confirmed as *Sarcomphalus joazeiro* (Mart.) Hauenschild, according to nomenclature validated by updated taxonomic platforms, such as World Flora Online and “Flora e Funga do Brasil".

The aqueous extract of *S. joazeiro* leaves was prepared by cold maceration, with adaptations from refs 15 and 16 Fresh leaves were washed, dried in an oven at 50 °C with air circulation for 24 h, and crushed until a fine powder was obtained. 50 g of powder was dispersed in 1 L of sterile ultrapure water and kept for 72 h under stirring. After that step, the extract was vacuum filtered twice using qualitative filter paper. The extract was frozen for 24 h and subsequently subjected to freeze-drying (−31 °C and 133.3 Pa), obtaining a greenish powder.

### Green Synthesis of ZnO Nanoparticles (ZnONPs)

The biosynthesis of zinc oxide nanoparticles (ZnONPs) was carried out based on the method described by Selim et al.^68^ with some modifications by substitution of the zinc nitrate hexahydrate precursor (Zn­(NO_3_)_2_·6H_2_O) by zinc acetate dihydrate (Zn­(CH_3_CO_2_)_2_·2H_2_O) and the use of anhydrous ethyl alcohol for purification.

As a first step, 2.5 mg of the lyophilized extract was dissolved in 25 mL of ultrapure water, resulting in a final concentration of 0.1 mg/mL. The solution was heated on a magnetic stirrer at 60 °C.

After stabilizing at 60 °C, 2.5 g of zinc acetate dihydrate was gradually added to the solution under intense stirring. The solution was kept under the same conditions for 1 h until the color changed to light yellow, indicating the progress of the reaction.

Purifying the resulting paste involved multiple washes with distilled water and anhydrous ethyl alcohol (3:1). Each wash cycle was followed by centrifugation at 5000 rpm for 10 min to remove impurities. This procedure was repeated until the washing solution became clear.

As a final step, the sample was calcined in a muffle furnace with controlled heating at a 5 °C/min rate until it reached 400 °C. The temperature was maintained for 2 h, forming white ZnONPs powder.

### Synthesis of PPy and ZnONPs@PPy Composites

For the synthesis of PPy, 35 μL of pyrrole was added to 25 mL of 1.0 M hydrochloric acid (HCl) aqueous solution, followed by stirring for 5 min for complete homogenization. A second solution containing the oxidizing agent was prepared by dissolving 0.082 g of ferric chloride (FeCl_3_) in 25 mL of 1.0 M HCl aqueous solution. The oxidizing solution was added dropwise to the pyrrole solution under continuous stirring, maintaining the system in reaction for 24 h at room temperature, while avoiding temperature fluctuations. After polymerization, the polypyrrole (PPy) was filtered, washed several times with distilled water to remove soluble residues, and dried under a vacuum at 60 °C for 15 h.

The composite of ZnONPs@PPy was prepared with an additional step, involving the addition of 50 mg of ZnONPs dispersion into the monomer solution. All of the steps remain the same, as previously described in the polymerization of PPy, allowing chemical polymerization by coating ZnO nanoparticles with PPy.

### Synthesis of PPy and ZnONPs@PPy Composites on Cotton Fibers and the Impregnation of ZnONPs into Cotton Fibers

Cleaned cotton fabric (5 cm × 5 cm) was immersed in the solution containing pyrrole, which was kept under stirring to provide the homogeneous impregnation of monomers into the cotton fibers. The following polymerization occurs according to previously reported steps. The impregnation favored by the adsorption of species into cotton fibers makes possible the direct polymerization of polypyrrole on cotton fibers that progressively acquire a dark aspect. Incorporating ZnONPs@PPy composites on cotton fibers follows the same steps as preparing the composite, with the cotton fiber immersed in the mixture of monomers and ZnONPs (50 mg).

The direct impregnation of ZnONPs into cotton fabrics was conducted as follows: previously washed and dried fabrics were immersed in a solution containing 20 mg/mL of ZnONPs. The system was immersed in an ultrasonic bath for 30 min, providing a uniform dispersion of the nanoparticles and their efficient adhesion to the fibers.

After this step, the fabrics were removed from the solution and dried at room temperature, preserving the cotton’s structural properties. The resulting material was stored under appropriate conditions for subsequent characterization and evaluation of its antibacterial properties.

### Characterization Techniques

The absorption spectra of zinc oxide nanoparticles (ZnONPs), polypyrrole (PPy), and zinc oxide nanoparticles coated by PPy (ZnONPs@PPy) were measured using a Hach DR500 UV–vis spectrophotometer in the range of 200 to 800 nm, with a step of 1 nm. The data obtained were treated according to the Tauc method.^69^ The structure of the synthesized materials was evaluated from Fourier transform infrared spectroscopy (FTIR) using the KBr method on a IRPrestige- 21 spectrometer (Shimadzu). The surface morphology of the samples under study was examined using a scanning electron microscope (SEM) model Vega 3XM Tescan. TEM images were carried out on Talos F200i S/TEM (ThermoFisher Scientific, Waltham, MA) operated at 200 keV. X-ray diffraction (XRD) analyses of the synthesized samples were conducted using a Miniflex powder diffractometer (Rigaku) equipped with a Cu–Kα radiation source (λ = 1.5406 Å), operating at 40 kV and 15 mA under continuous scanning conditions. Data were collected over a 2θ range of 20–90°, with a scan speed of 10°/min and a step size of 0.02°, all at room temperature. Photoluminescence assays were conducted with ZnO-based samples (conventional ZnONPs-C) and green-synthesized structures (ZnONPs-G), excited by a UV longwave ultraviolet source (365 nm), with a reflection mode acquired emission data through an optical fiber (diameter of 100 μm) connected to a spectrometer (Ocean Optics USB4000).

### Preparation of Standards for HPLC/DAD Analysis

Standard solutions for the seven investigated phenolic acids and flavonoids (Sigma-Aldrich) were prepared: caffeic acid, p-coumaric acid, ferulic acid, cinnamic acid, naringenin, pinocembrin, and apigenin. The solutions were prepared by dissolving the standards in HPLC-grade methanol (Agilent Technologies, Germany) to produce 1000 ppm stock solutions, which were then used to prepare concentrations ranging from 1 to 50 ppm. The absorbance of the 20 ppm standards of each phenolic acid and flavonoid was measured at wavelengths 190 to 400 nm to find the optimal wavelength for HPLC-DAD measurements.

### HPLC/DAD Analysis

HPLC analysis of *S. joazeiro* extract was performed by comparing it with reference standards of phenolic acids and flavonoids using an Agilent 1260 HPLC system (Agilent Tech., Germany) equipped with a UV–vis DAD. The separation was conducted by the gradient method using a C18 chromatographic column (250 mm × 4.0 mm × 5 μm, Macherey-Nagel, Germany). The phenolic standard solutions and mixtures were injected into the system using an autoinjector. The mobile phase consisted of a mixture of solvents A (ultrapure water) and B (methanol: acetonitrile, 60:40, HPLC grade, Agilent Tech., Germany), both acidified with 0.1% formic acid (Sigma-Aldrich) using the following elution gradient: 0–15 min: 15% B in A; 17 min: 40% B in A; 30 min: 30% B in A, 38 min: 15% B in A, maintaining this composition for 45 min. The wavelengths used were 310 nm for caffeic acid, p-coumaric acid, and ferulic acid; 290 nm for cinnamic acid, naringenin, and pinocembrin; and 340 nm for apigenin. The mobile phase flow rate was 0.5 mL/min, and the injection volume was 20 μL. The mobile phases, solutions, and samples were filtered through a Millipore membrane filter with a filter diameter of 13 mm and a pore diameter of 0.22 μm (Millipore). The samples were dissolved in HPLC-grade methanol (30 mg/mL). Quantification was performed by integrating the peaks using the external standard method. The analyses were conducted at room temperature and in triplicate, and the peaks were confirmed by comparing their retention time with those of the reference standards and by DAD spectra (190 to 400 nm). The quantifications of the compounds were based on analytical curves of the reference standards. The limit of detection (LOD) and the limit of quantification (LOQ) were calculated based on the standard deviation of the responses and the slope using three independent analytical curves. LOD and LOQ were calculated as 3.3 and 10 σ/S, respectively, in which σ is the standard deviation of the response, and S is the slope of the calibration curve.

### Antibacterial Activity Assays

The tests were carried out with strains of *S. aureus* (ATCC 25923) and *E. coli* (ATCC 25922) provided by the UNIVASF Immunology and Microbiology Laboratory. The strains were maintained in BHI medium and 80% glycerol, stored at −20 °C. Replacements were performed 24 h before the assays to ensure viability and purity.

Bacterial suspensions were standardized based on optical density (OD) using a UV–vis spectrophotometer operating at a single wavelength of 620 nm. The objective was to adjust the cell concentration to values previously established as equivalent to 1.5 × 10^8^ colony-forming units per milliliter (CFU mL^–1^) from bacterial cultures in the exponential growth phase.

The broth microdilution test was carried out according to the guidelines of (CLSI, 2019) to determine the Minimum Inhibitory Concentration (MIC) and Minimum Bactericidal Concentration (MBC) of ZnONPs, PPy, and ZnONPs@PPy aqueous dispersion, in which a predetermined content of sample was sonicated for 30 min. In a 96-well microplate, 100 μL of sterile TSB broth and 100 μL of the solutions under study were added, followed by serial dilutions. Ten μL of bacterial suspensions (1.5 × 10^5^ CFU mL^–1^) were added to the wells and incubated at 37 °C for 24 h. After incubation, aliquots of triphenyl tetrazolium hydrochloride (CTT) were added to all wells to determine the MIC by color change. For MBC, aliquots of the solutions were seeded in a TSA medium and incubated again. Negative (TSB broth) and positive (TSB broth plus bacterial suspension) controls were used with the assays performed in triplicate.

For agar diffusion assays, 5 mm diameter cotton disks were used. These disks were previously impregnated with zinc oxide nanoparticles (ZnONPs), polypyrrole (PPy), and polypyrrole with zinc oxide nanoparticles (ZnONPs@PPy). Bacterial suspensions of *E. coli* and *S. aureus* (1.5 × 10^8^ CFU mL^–1^) were seeded in Petri dishes containing TSA agar. Cotton fabric disks without antibacterial agents were used as a negative control. The fabric samples were impregnated with ZnONPs, PPy, and ZnONPs@PPy and applied aseptically to the plates with the bacteria. The plates were incubated at 37 °C for 24 h to observe the formation of inhibition zones. The diameters of the zones were measured on a black background to ensure better contrast and measurement accuracy. Each experiment was performed three times on different days. The results were expressed as mean ± standard deviation.

The kinetics of the death time curve was determined by evaluating the interaction of bacterial suspensions of *E. coli* and *S. aureus* (1.5 × 10^6^ CFU mL^–1^) in TSB broth with lyophilized antibacterial nanoparticles under distinct conditions (under dark and sunlight direct incidence). Positive control samples were treated with sunlight as a control to evaluate the isolated effect of radiation. For the evaluation of the influence of the sun on the antibacterial activity of compounds, the ZnONPs and ZnONPs@PPy samples were exposed to sunlight radiation in an open-air area in the city of Juazeiro, Bahia, Brazil, from 12:00 pm to 3:00 pm. At 10, 20, 30, 60, 120, and 180 min intervals, 100 μL aliquots of the resulting suspension were inoculated into Petri dishes and incubated at 37 °C for 24 h. In parallel, aliquots of ZnONPs, PPy, and ZnONPs@PPy samples, under dark conditions, were inoculated in Petri dishes with TSA medium. All treatments were performed in triplicate. After the incubation period, colonies were counted by direct inspection to determine the number of viable cells in each treatment.

The surface disinfection test used sterilized square stainless-steel plates (5 × 5 cm). The surfaces of the plates were rubbed with 100 μL aliquots of bacterial suspensions containing 1.5 × 10^8^ CFU mL^–1^. Then, the plates were cleaned with different types of fabric (5 cm × 5 cm) impregnated with nanoparticles and nanocomposites (ZnONPs, PPy, and ZnONPs@PPy). After 2 min of the cleaning procedure, the stainless-steel plates containing residual microorganisms were stamped onto the surface of the TSA agar medium. The plates were incubated at 37 °C for 24 h. After incubation, bacterial growth was observed and quantified by directly examining the colonies formed on the agar medium, with the assay performed in triplicate.

The inhibition of biofilm formation was investigated using *S. aureus* strains, widely recognized for their ability to form biofilms, and *E. coli* strains. Initially, bacterial suspensions were prepared in trypticase soy broth (TSB) with the addition of 25% glucose, with a concentration of 1.5 × 10^5^ CFU mL^–1^.

For the treatments, cotton fabric samples (5 cm × 5 cm) previously impregnated with zinc oxide nanoparticles (ZnONPs), polypyrrole (PPy), or (ZnONPs@PPy) were used. The samples were added to test tubes containing the corresponding bacterial suspensions and divided into two experimental groups: one group was exposed to sunlight for 60 min before incubation, and the other was kept in the dark. In parallel, controls containing 10 mL of TSB, bacteria, and cotton fabric without the addition of antibacterial agents were prepared, both in light and dark conditions. After preparation, all tubes were incubated at 37 °C for 24 h.

After incubation, the contents of all tubes were discarded, and the tissue samples were transferred to sterile tubes. The tissues were washed thrice with 10 mL of sterile distilled water, with the liquid discarded after each wash. Subsequently, the tubes with the tissues were left to dry at room temperature for 5 min and inverted on paper towels.

Then, 10 mL of saline solution was added to the tubes. Then, the tubes were sonicated in an ultrasonic bath at 40 kHz for 15 min to remove the cells adhered to the walls of the tubes and tissue samples. After sonication, the bacterial suspension was diluted to 1:100 in a saline solution. Aliquots of 100 μL of the resulting suspension were collected in triplicate from each system and plated on tryptic soy agar (TSA). After plating, the plates were incubated at 37 °C for 24 h. At the end of the incubation, bacterial growth was assessed by directly examining the colonies formed after resuspension of the viable biofilm cells.

The quantification of cultivable biofilm cells was calculated from the antibacterial rate:

$$\mathrm{antibacterial}\;\mathrm{activity}\;(\%)=\frac{N_{\mathrm{control}}-N_{\mathrm{sample}}}{N_{\mathrm{control}}}\times 100\%$$ 3

*N*_control_ is the mean number of bacteria in the tissue sample without antimicrobial species. *N*_sample_ is the mean number of bacteria in the tissue samples modified with the tested nanomaterials (CFU per sample). Assays are performed in triplicate.

### Nitroblue Tetrazolium (NBT) Reduction Assay for Reactive Oxygen Species (ROS) Assessment

The potential for reactive oxygen species (ROS) generation was assessed by the nitroblue tetrazolium (NBT) reduction assay. The culture medium was prepared with 4% (w/v) agar, sterilized by autoclaving, cooled, and supplemented with 0.1% (w/v) NBT in a 1:1 (v/v) ratio. The mixture was poured into 12-well plates, allowed to solidify, and then drilled to create 5 mm-diameter wells. A suspension of *E. coli* (1 × 10^7^ CFU/mL) was mixed with the ZnONPs, PPy, and ZnONPs@PPy samples in a 1:1 (v/v) ratio, totaling 200 μL, with a final concentration of the samples at 4x MBC.

The suspensions were preincubated for 20 min. Then, 100 μL of each mixture was added to the wells. The negative control consisted of a mixture of saline solution (0.85%) and bacterial suspension (1:1, v/v), and a second control contained saline solution supplemented with PPy at 4x MBC. The positive control consisted of a hydrogen peroxide solution (1.5%). ROS generation was inferred by formazan formation, evidenced by purple halos around the wells, with the intensity and diameter used as indicators of sample activity.

## Supplementary Material



## Notes

Table S1: quantification by HPLC/DAD of phenolic acids and flavonoids present in the aqueous extract of *Sarcomphalus joazeiro* leaves; Figure S1: general scheme for green reduction of ZnONPs exploring ferulic acid as reducing agent; Figure S2: EDS map for ZnONPs@PPy compound; Figure S3: hydrodynamic diameter and polydispersity index (PDI) of the particles by dynamic light scattering (DLS); (A) ZnONPs; (B) PPy; and (C) ZnONPs@PPy composite; Figure S4: photoluminescence spectra of ZnO nanoparticles synthesized by conventional route (ZnONPS-C) and alternative (green synthesis–ZnONPs-G); Table S2: values for MIC and MBC of conventional and green ZnONPs; Figure S5: photo of wells with agar (a) negative control, (b) pure polypyrrole in the absence of *E. coli*, (c) positive control (H2O2), (d) ZnO, (e) PPy, and (f) ZnONPs@PPy; Figure S6: control experiments with ciprofloxacin (0.1 mg/mL), against *S. aureus* (a) and *E. coli* (b); Figure S7: images of Petri dishes showing remaining colonies of *E. coli* and *S. aureus* after the surface disinfection test; bacteria were transferred by direct contact (stamping) from stainless steel plates, previously sanitized with pure cotton fabrics (control) and cotton fabrics impregnated with ZnONPs, PPy, and ZnONPs@PPy; Figure S8: sunlight spectrum in Juazeiro-BA-Brazil (between 12 and 3 pm); Figure S9: SEM images of pure cotton fabrics and fabrics impregnated with ZnONPs, PPy, and ZnONPs@PPy after 24 h of exposure to *S. aureus*; (A) pure cotton after contact with *S. aureus*; (B) cotton impregnated with ZnONPs; (C) cotton impregnated with PPy; (D) cotton impregnated with ZnONPs@PPy; Figure S10: SEM images of pure cotton fabrics and fabrics impregnated with ZnONPs, PPy, and ZnONPs@PPy after 24 h of exposure to *E. coli*; (A) pure cotton after contact with *E. coli*; (B) cotton impregnated with ZnONPs; (C) cotton impregnated with PPy; (D) cotton impregnated with ZnONPs@PPy; Table S3: comparison of properties and applications of ZnO nanoparticles prepared with different precursors (PDF)
