# Supplementary material for: Light-Mediated Antibacterial Activity of Composites of Polypyrrole and Green Zinc Oxide Nanoparticles Synthesized using Sarcomphalus joazeiro Extract
Source: ACS Omega. 2025 Sep 20;10(38):43591–606. doi: 10.1021/acsomega.5c03456 (PMC12489726; doi:10.1021/acsomega.5c03456)
Supplement: Supplementary file 1 [file ao5c03456_si_001.pdf]

Supporting Information

Light-mediated antibacterial activity of composites of polypyrrole and green zinc oxide nanoparticles synthesized using *Sarcomphalus joazeiro* extract

Milena L. Guimarães[1,2], Ricardo F. Ferraz[1], Raquel A. P. Oliveira[1], José Galberto M. da Costa[3], Débora Odília D. Leite[3], Mateus M. da Costa[1], Helinando P. de Oliveira*[1,2]

[1]Institute of Materials Science, Universidade Federal do Vale do São Francisco, Avenida Antônio Carlos Magalhães, 510 - Santo Antônio CEP: 48902-300 – Juazeiro, Bahia, Brazil.
[2]RENORBIO - Northeast Biotechnology Network, Universidade Federal Rural de Pernambuco (UFRPE), Recife, Pernambuco, Brazil.
[3]Natural Products Research Laboratory, Universidade Regional do Cariri, Coronel Antônio Luíz Street, 1161–Pimenta, Crato 63105-010, Ceará, Brazil.

*Corresponding author:
E-mail address: helinando.oliveira@univasf.edu.br (Helinando P. de Oliveira)

Tauc method

The Tauc method postulates that the absorption coefficient depends on the energy α and can be expressed by the following eq. S1[1]:

$$(\alpha h\upsilon)^2 = k(h\upsilon - E_g) \quad \text{(S1)}$$

where υ represents the photon frequency, h is Planck's constant, Eg is the band gap energy, K is a constant, α is the absorption coefficient given in terms of the absorbance A and the optical path (d), according to eq. S2:

$$\alpha(\lambda) = \frac{2.303}{d}A(\lambda) \quad \text{(S2)}$$

The band gap energy (E_g) of different nanomaterials can be calculated by evaluating the graphs of $(\alpha h\upsilon)^2$ versus the photon energy ($h\upsilon$), where the band gap energy is the value found in the extrapolation of the straight line to $(\alpha h\upsilon)^2 = 0$. [2,3]

Table S1. Quantification by HPLC/DAD of phenolic acids and flavonoids present in the aqueous extract of *Sarcomphalus joazeiro* leaves.

Compound	LD (mg/mL)	LQ (mg/mL)	***Sarcomphalus joazeiro***	
			mg/g	%
Caffeic acid	0.0001	0.0003	0.0233 ± 0.002887	0.00233
p-Coumaric acid	0.0007	0.0025	0.0526 ± 0.016166	0.00526
Ferulic acid	0.0048	0.0162	0.2410 ± 0.015588	0.02410
Cinnamic acid	00009	0.0032	0.0913 ± 0.001155	0.00913
Naringenin	0.0011	0.0036	Nd	nd
Pinocembrin	0.0030	0.0102	Nd	nd
Apigenin	0.0027	0.0092	Nd	nd
**Total**			0.4082	0.04082

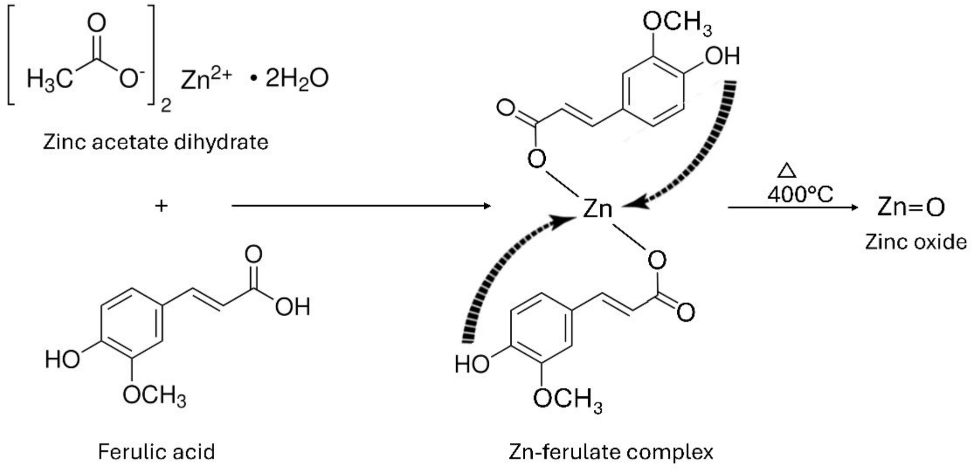

Figure S1. General scheme for green reduction of ZnONPs exploring ferulic acid as reducing agent.

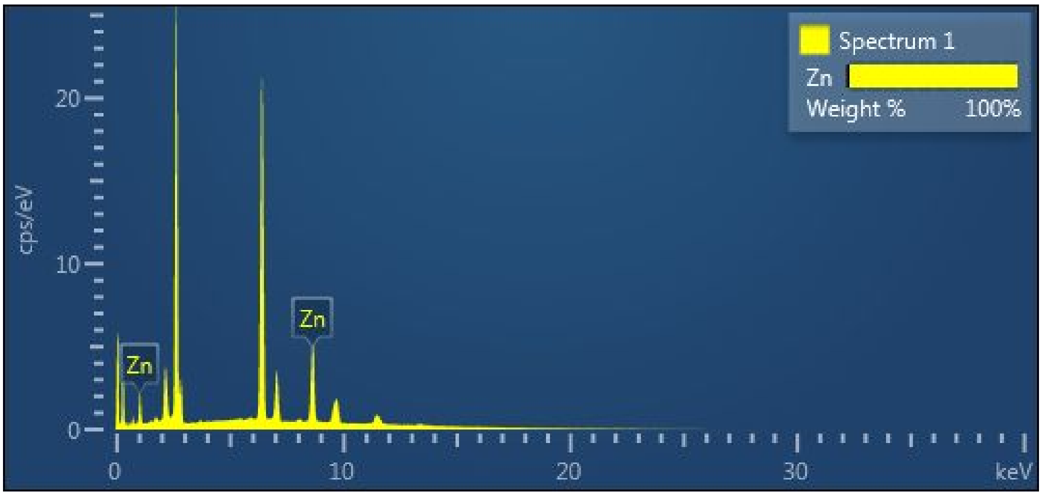

Figure S2. EDS map for ZnONPs@PPy compound.

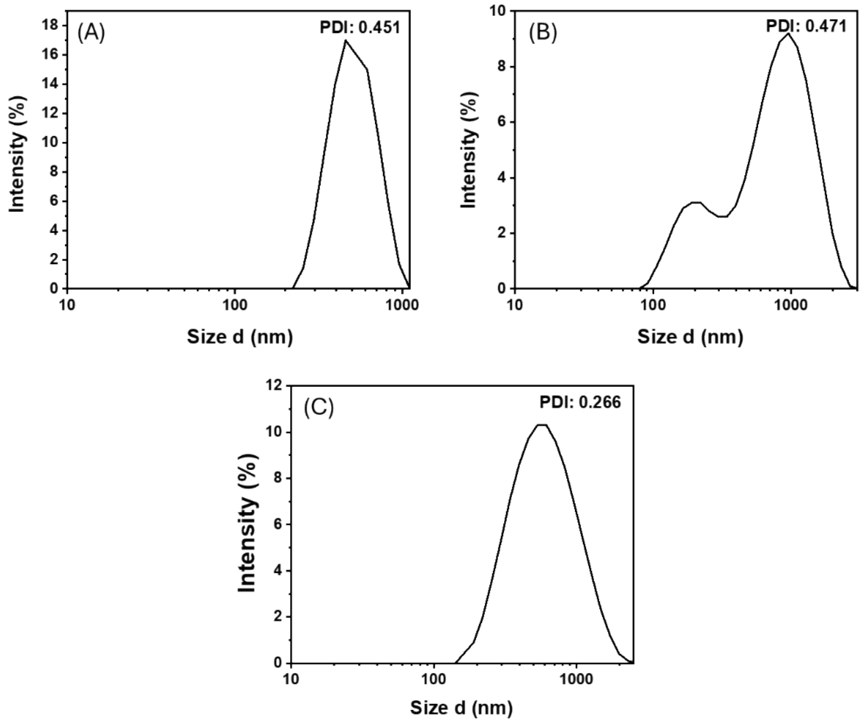

Figure S3. Hydrodynamic diameter and polydispersity index (PDI) of the particles by dynamic light scattering (DLS): (A) ZnONPs; (B) PPy; and (C) ZnONPs@PPy composite.

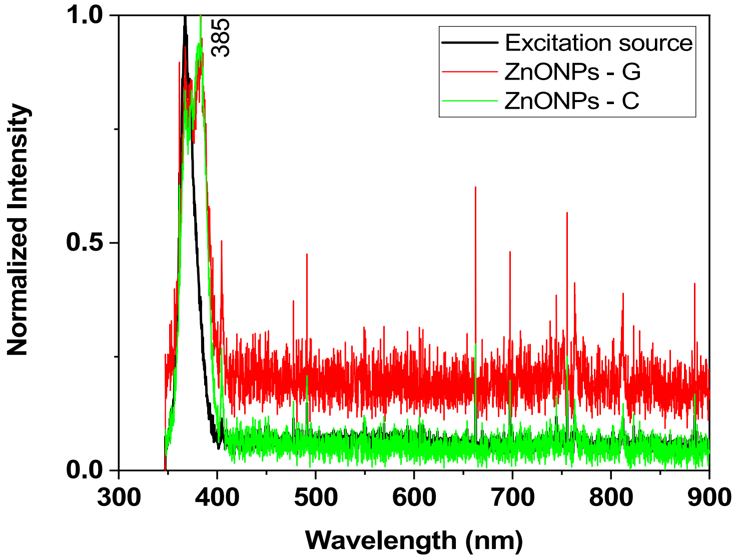

Figure S4. Photoluminescence spectra for ZnO nanoparticles synthesized through the conventional route (ZnONPS – C) and the alternative (green synthesis – ZnONPs-G).

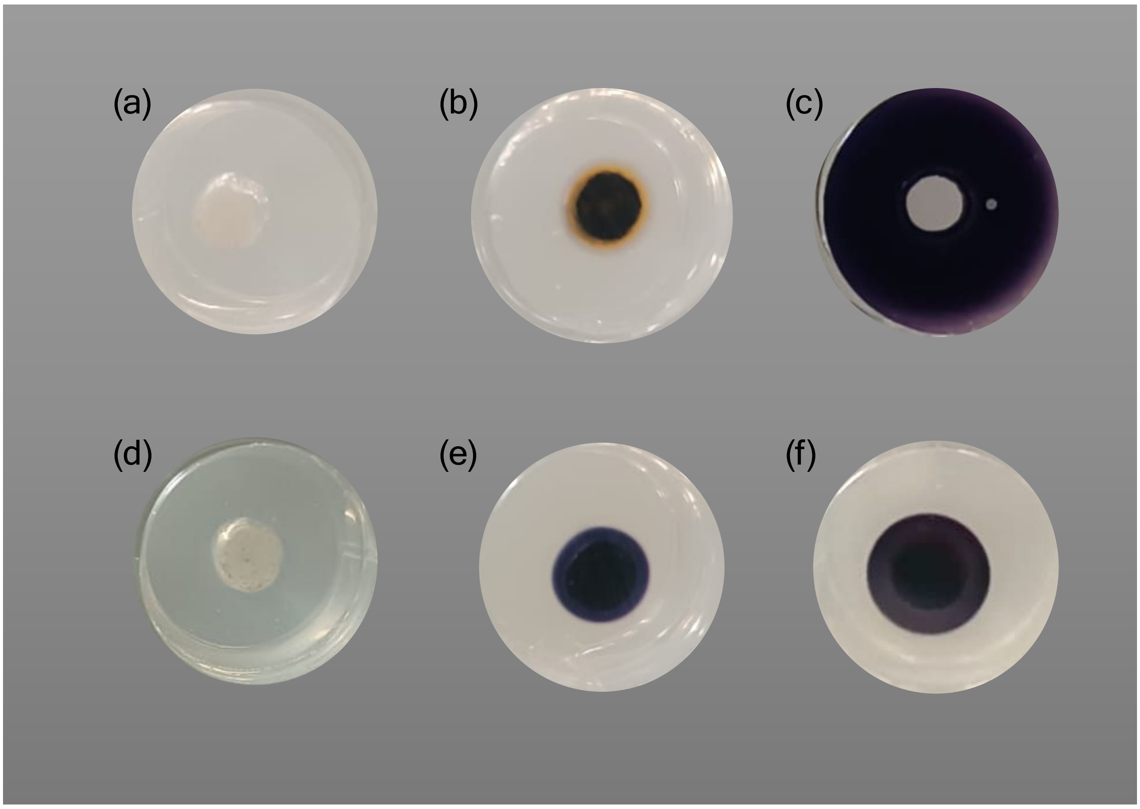

Figure S5. Photo of wells with agar and a central zone filled with (a) negative control, (b) pure polypyrrole in the absence of *E. coli*, (c) positive control (H_2O_2), (d) ZnO, (e) PPy, and (f) ZnONPs@PPy

Chemical Synthesis of Zinc Oxide Nanoparticles (ZnONPs-C)

ZnO nanoparticles were synthesized chemically using zinc nitrate hexahydrate [$Zn(NO_3)_2 \cdot 6H_2O$] (Exodo Científica) as a metal precursor, anhydrous ethylene glycol (Sigma-Aldrich, 99.8%) as a solvent, polyvinyl alcohol (Sigma-Aldrich, 99.8%) as a dispersing agent, and sodium hydroxide (NaOH, Dinâmica, 99%) as a precipitating agent, according to the methodology of Biron et al. [1] As a first step, 25 mL of a 1 $mol \cdot L^{-1}$ NaOH solution in ethylene glycol was slowly added dropwise to 25 mL of a 0.5 $mol \cdot L^{-1}$ $Zn(NO_3)_2 \cdot 6H_2O$ solution, also in ethylene glycol, under continuous stirring. Next, 0.05 g of PVA was added to the reaction mixture to stabilize the colloidal system. The resulting solution was heated under reflux at 140°C for 4 hours. After the reaction, the product was separated by centrifugation at 5,000 rpm for 15 minutes, followed by successive washes with ethyl alcohol to remove impurities. The resulting solid material was oven-dried at 80°C for 24 hours for the following characterizations.

Table S2. Values for MIC and MBC of conventional and green ZnONPs

	Conventional ZnONPs	
	**MIC**	**MBC**
S. aureus	250 µg/mL	500 µg/mL
E. coli	1000 µg/mL	2000 µg/mL

	Green ZnONPs	
	**MIC**	**MBC**
S. aureus	500 µg/mL	500 µg/mL
E. coli	2000 µg/mL	2000 µg/mL

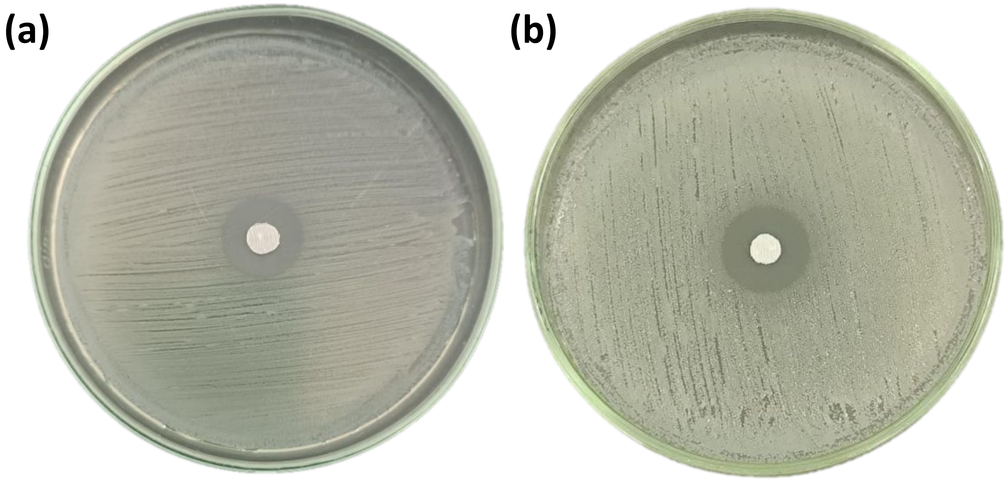

Figure S6. Control experiments with ciprofloxacin (0.1 mg/mL), against *S. aureus* (a) and *E. coli* (b).

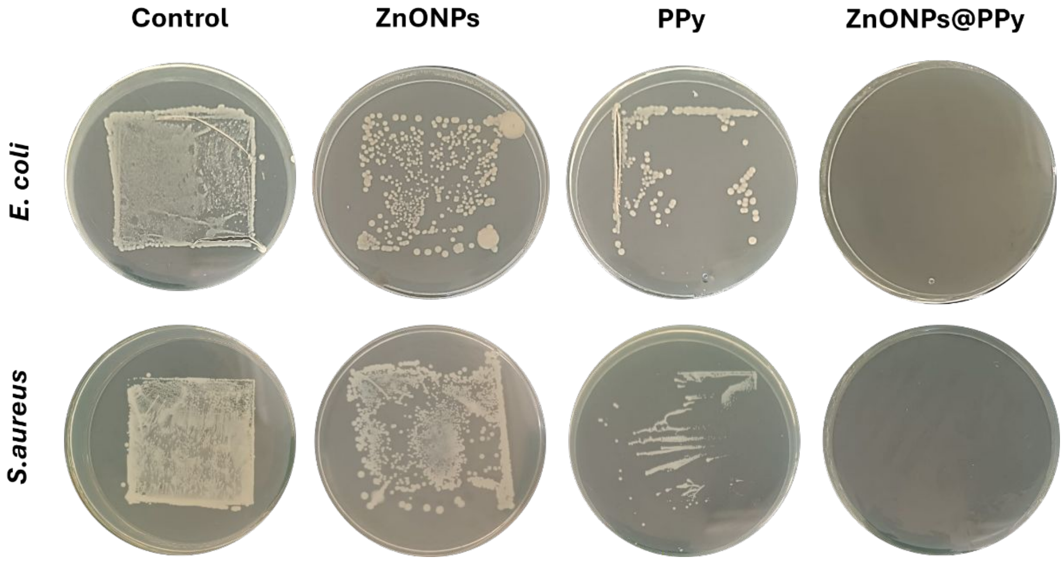

Figure S7. Images of Petri dishes showing remaining colonies of *E. coli* and *S. aureus* after the surface disinfection test. The bacteria were transferred by direct contact (stamping) from stainless steel plates, previously sanitized with pure cotton fabrics (control) and cotton fabrics impregnated with ZnONPs, PPy, and ZnONPs@PPy.

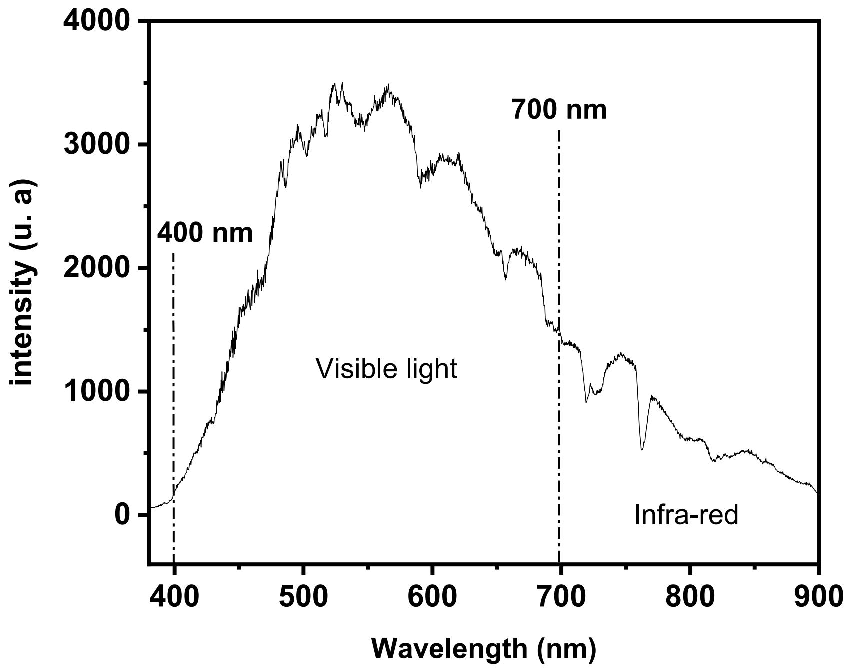

Figura S8. Sunlight spectrum in Juazeiro-BA-Brazil (between 12 pm and 3 pm).

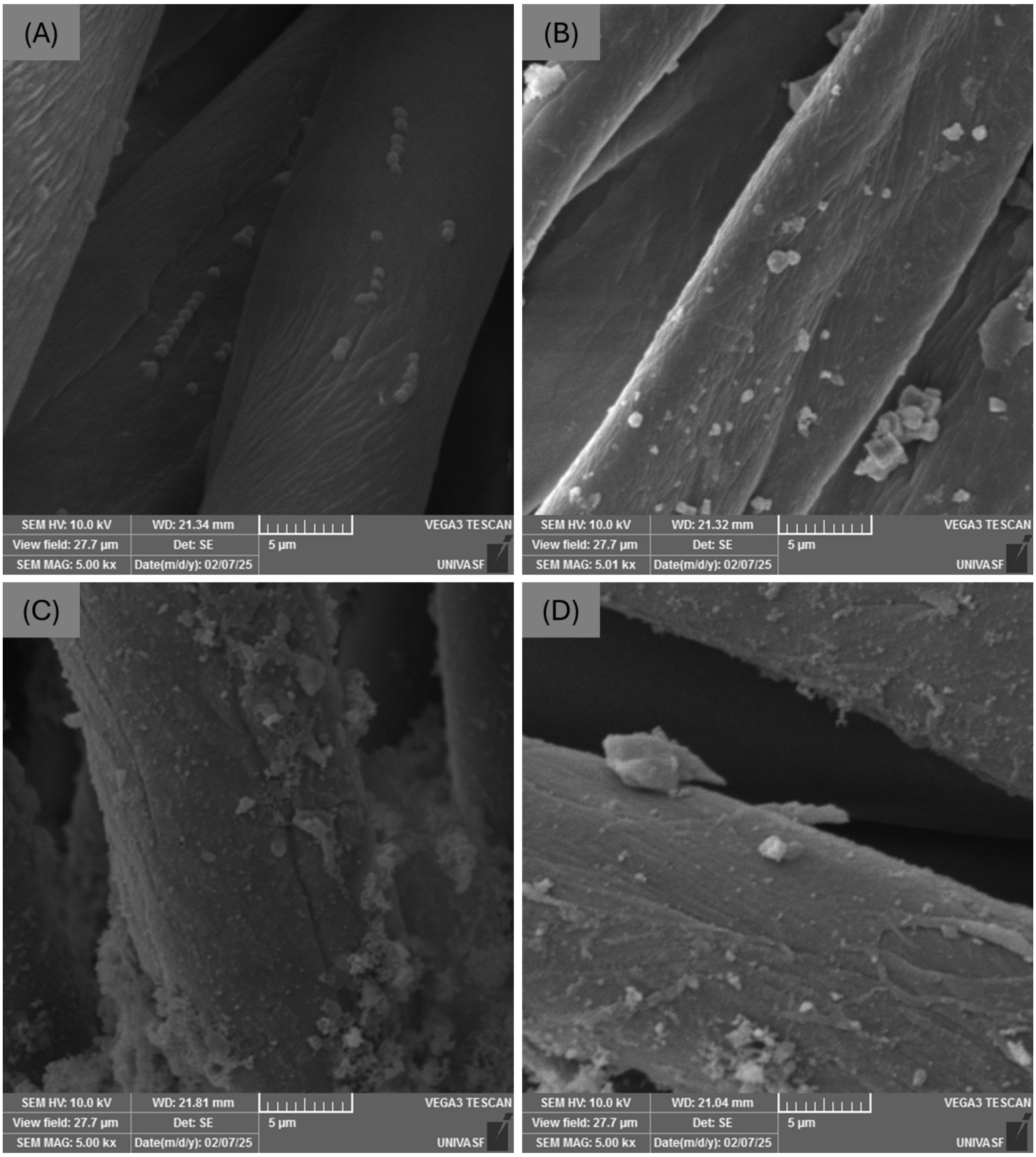

Figure S9. SEM images of pure cotton fabrics and fabrics impregnated with ZnONPs, PPy, and ZnONPs@PPy after 24 h of exposure to *S. aureus*. (A) Pure cotton after contact with *S. aureus*; (B) Cotton impregnated with ZnONPs; (C) Cotton impregnated with PPy; (D) Cotton impregnated with ZnONPs@PPy.

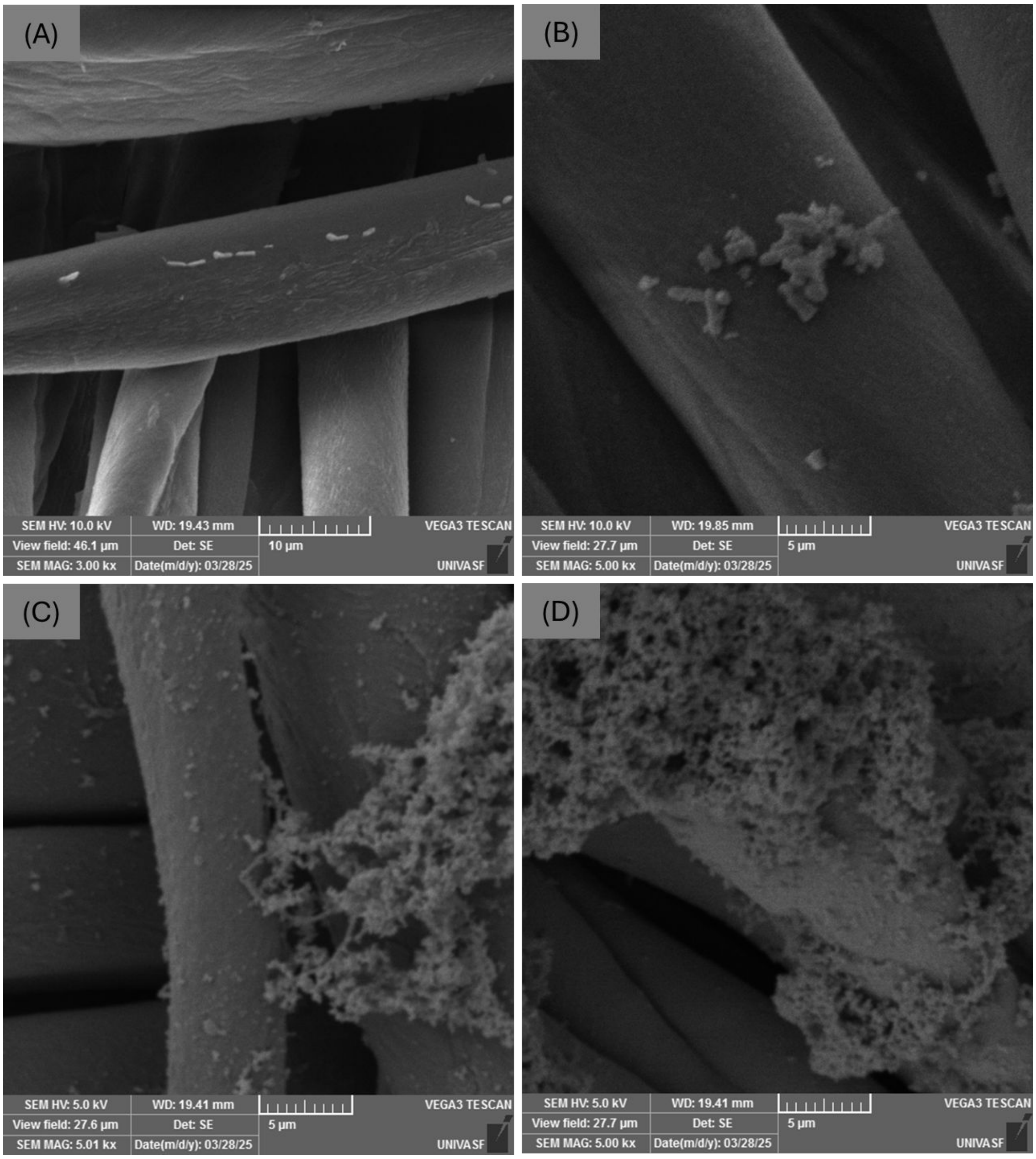

Figure S10. SEM images of pure cotton fabrics and fabrics impregnated with ZnONPs, PPy, and ZnONPs@PPy after 24 h of exposure to *E. coli*. (A) Pure cotton after contact with *E. coli*; (B) Cotton impregnated with ZnONPs; (C) Cotton impregnated with PPy; (D) Cotton impregnated with ZnONPs@PPy.

Table S3. Comparison of properties and applications of ZnO nanoparticles prepared with different precursors.

Precursor	Extract	UV-vis	Size of particles	Zeta potential	Antibacterial activity	Ref.
Zinc acetate dihydrate	*Buchanania obovata*	340 nm and 320 nm	40 nm and 25 nm	-36.7 mV and - 45.5 mV	*E. coli* and *S. aureus*	5
Zinc acetate dihydrate	fungal extract of *Aspergillus sp*	280 nm and 340 nm	28.9 nm	-18.16 mV	*E. coli, P. aeruginosa, S. aureus, B. subtilis, C. albicans,* and *A. flavus*	6
Zinc nitrate hexahydrate	Propolis	280 nm	1601 nm and 1751 nm	-11.8 mV and -27.1 mV	*E. coli, S. typhi, and P. aeruginosa, B. cereus,* and *S. aureus*	7
Zinc acetate dihydrate	*Rhus coriaria*	359 nm	20.51 nm	- 19.9 mV	-	8
Zinc acetate dihydrate	*Tecoma stans*	345 nm	1.5 nm	-27 mV	*S. aureus* and *E. coli*	9
Zinc acetate dihydrate	*Fumaria officinalis* and *Peganum harmala*	294 nm and 303 nm	19.55 nm and 25.10 nm	-	*S. aureus* and *C. michiganensis*	10
Zinc acetate	*Ziziphus jujuba*	-	90 nm	+11.9 mV	*E. coli* and *S. aureus*	11
Zinc nitrate hexahydrate	*Ziziphus* leaf	367 nm	44.63 nm	-	*S. aureus* and *P. aeruginosa*	12
Zinc acetate dihydrate	*Sarcomphalus joazeiro*	368 nm	24.47 ± 6.80 nm	-11.6 ± 1.91 mV	*E. coli* and *S. aureus*	This work

References

(1) Makuła, P.; Pacia, M.; Macyk, W. How To Correctly Determine the Band Gap Energy of Modified Semiconductor Photocatalysts Based on UV-Vis Spectra. *Journal of Physical Chemistry Letters* **2018**, *9* (23), 6814–6817. https://doi.org/10.1021/acs.jpclett.8b02892.

(2) Guimarães, M. L.; da Silva, F. A. G.; da Costa, M. M.; de Oliveira, H. P. Coating of Conducting Polymer-Silver Nanoparticles for Antibacterial Protection of Nile Tilapia Skin Xenografts. *Synth Met* **2022**, *287* (February). https://doi.org/10.1016/j.synthmet.2022.117055.

(3) Sadrolhosseini, A. R.; Rashid, S. A.; Noor, A. S. M.; Kharazmi, A.; Lim, H. N.; Mahdi, M. A. Optical Band Gap and Thermal Diffusivity of Polypyrrole-Nanoparticles Decorated Reduced Graphene Oxide Nanocomposite Layer. **2016**, *2016*.

(4) Biron, D. da S.; Santos, V. dos; Bergmann, C. P. Synthesis and Characterization of Zinc Oxide Obtained by Combining Zinc Nitrate with Sodium Hydroxide in Polyol Medium. *Materials Research* **2020**, *23* (2). https://doi.org/10.1590/1980-5373-mr-2020-0080.

(5) Jabbar, K. Q.; Barzinjy, A. A. Biosynthesis and Antibacterial Activity of ZnO Nanoparticles Using Buchanania Obovata Fruit Extract and the Eutectic-Based Ionic Liquid. *Nanotechnology* **2024**, *35* (26). https://doi.org/10.1088/1361-6528/ad375e.

(6) Abdelrahman, S. E. S. A. H.; El Hawary, S.; Mohsen, E.; El Raey, M. A.; Selim, H. M. R. M.; Hamdan, A. M. E.; Ghareeb, M. A.; Hamed, A. A. Bio-Fabricated Zinc Oxide Nanoparticles Mediated by Endophytic Fungus Aspergillus Sp. SA17 with Antimicrobial and Anticancer Activities: In Vitro Supported by in Silico Studies. *Front Microbiol* **2024**, *15*. https://doi.org/10.3389/fmicb.2024.1366614.

(7) Salama, S. A.; Essam, D.; Tagyan, A. I.; Farghali, A. A.; Khalil, E. M.; Abdelaleim, Y. F.; Hozzein, W. N.; Mubarak, M.; Nasr, F. A.; Eweis, A. A.; Al-Zharani, M.; Mahmoud, R. Novel Composite of Nano Zinc Oxide and Nano Propolis as Antibiotic for Antibiotic-Resistant Bacteria: A Promising Approach. *Sci Rep* **2024**, *14* (1). https://doi.org/10.1038/s41598-024-70490-8.

(8) Mongy, Y.; Shalaby, T. Green Synthesis of Zinc Oxide Nanoparticles Using Rhus Coriaria Extract and Their Anticancer Activity against Triple-Negative Breast Cancer Cells. *Sci Rep* **2024**, *14* (1). https://doi.org/10.1038/s41598-024-63258-7.

(9) Nivedha, K.; Kalaiarasi, K.; Poonkothai, M.; Swathilakshmi, A. V. Multifunctional Finishing of Cotton Fabric Using Zinc Oxide Nanoparticles Synthesized by Bio-Reduction Method. *Biomass Convers Biorefin* **2024**. https://doi.org/10.1007/s13399-024-05761-5.

(10) Hayat, K.; Din, I. U.; Alam, K.; Khan, F. U.; Khan, M.; Mohamed, H. I. Green Synthesis of Zinc Oxide Nanoparticles Using Plant Extracts of Fumaria Officinalis and Peganum Harmala and Their Antioxidant and Antibacterial Activities. *Biomass Convers Biorefin* **2024**. https://doi.org/10.1007/s13399-024-05804-x.

(11) Alharthi, M. N.; Ismail, I.; Bellucci, S.; Jaremko, M.; Abo-Aba, S. E. M.; Abdel Salam, M. Biosynthesized Zinc Oxide Nanoparticles Using Ziziphus Jujube Plant Extract Assisted by Ultrasonic Irradiation and Their Biological Applications. *Separations* **2023**, *10* (2). https://doi.org/10.3390/separations10020078.

(12) Habeeb, S. A.; Hammadi, A. H.; Abed, D.; Al-Jibouri, L. F. Green Synthesis of Metronidazole or Clindamycin-Loaded Hexagonal Zinc Oxide Nanoparticles from Ziziphus Extracts and Its Antibacterial Activity. *Pharmacia* **2022**, *69* (3), 855–864. https://doi.org/10.3897/pharmacia.69.e91057.
